# Information Security with Smart Hydrogels: Photo-Patterning and Multi-Stimuli Responsive Structural Color

**DOI:** 10.1007/s40820-026-02130-x

**Published:** 2026-03-31

**Authors:** Xiaoyu Guo, Ying Li, Farzana Hanif, Linhai Zhu, Miao Kong, Shufen Zhang, Yuang Zhang, Bingtao Tang

**Affiliations:** State Key Laboratory of Fine Chemicals, Frontiers Science Center for Smart Materials Oriented Chemical Engineering, Dalian University of Technology, Dalian, 116024 People’s Republic of China

**Keywords:** Structural color, Anti-opal hydrogel, Light-induced crosslinking, Information security

## Abstract

Photonically structured colors, characterized by high resolution and dynamic responsiveness, hold promising prospects in the field of information security. However, conventional patterning methods are often limited by high equipment costs and monotonous color outputs, which restrict their widespread adoption. To address these issues, this paper proposes a novel multi-color patterning method based on light-induced chemical crosslinking. By introducing light-initiated crosslinking molecules into anti-opal hydrogels, we developed a film that can be further regulated by photo-curing, enabling a “film formation first, then patterning” approach. The structural color hydrogels created using this method can display multi-color patterns, with a minimum line width of 15 μm, significantly enhancing their information-carrying capacity. Moreover, ultraviolet radiation can increase the degree of cross-linking, thereby inhibiting swelling behavior, enhancing tensile strength, reducing elongation at break, and causing the color of the inverse opal structure to shift toward blue or disappear. With inherent responsiveness to stress, temperature, and solvents, this approach enables dynamic information display and has excellent stability (able to cycle stably for more than 100 times). This work introduces a new method for patterning stimulus-responsive structural colors and opens up new possibilities for their use in applications such as ink-free printing, information encryption, and anti-counterfeiting.

**Supplementary Information:**

The online version contains supplementary material available at 10.1007/s40820-026-02130-x.

## Highlights

- A light-induced crosslinking strategy enables multi-color patterning of anti-opal hydrogel with a minimum line width of 15 μm, significantly enhancing information capacity.
- Control film crosslinking degree via UV irradiation allows adjust swelling, mechanical and optical properties of various stimulus-responsive structural color films simultaneously.
- A "film formation first, then patterning" approach provides a new train of thought for dynamic information encryption, security and ink-free printing.

**Supplementary Information:**

The online version contains supplementary material available at 10.1007/s40820-026-02130-x.

## Graphical abstract

**Figure:**
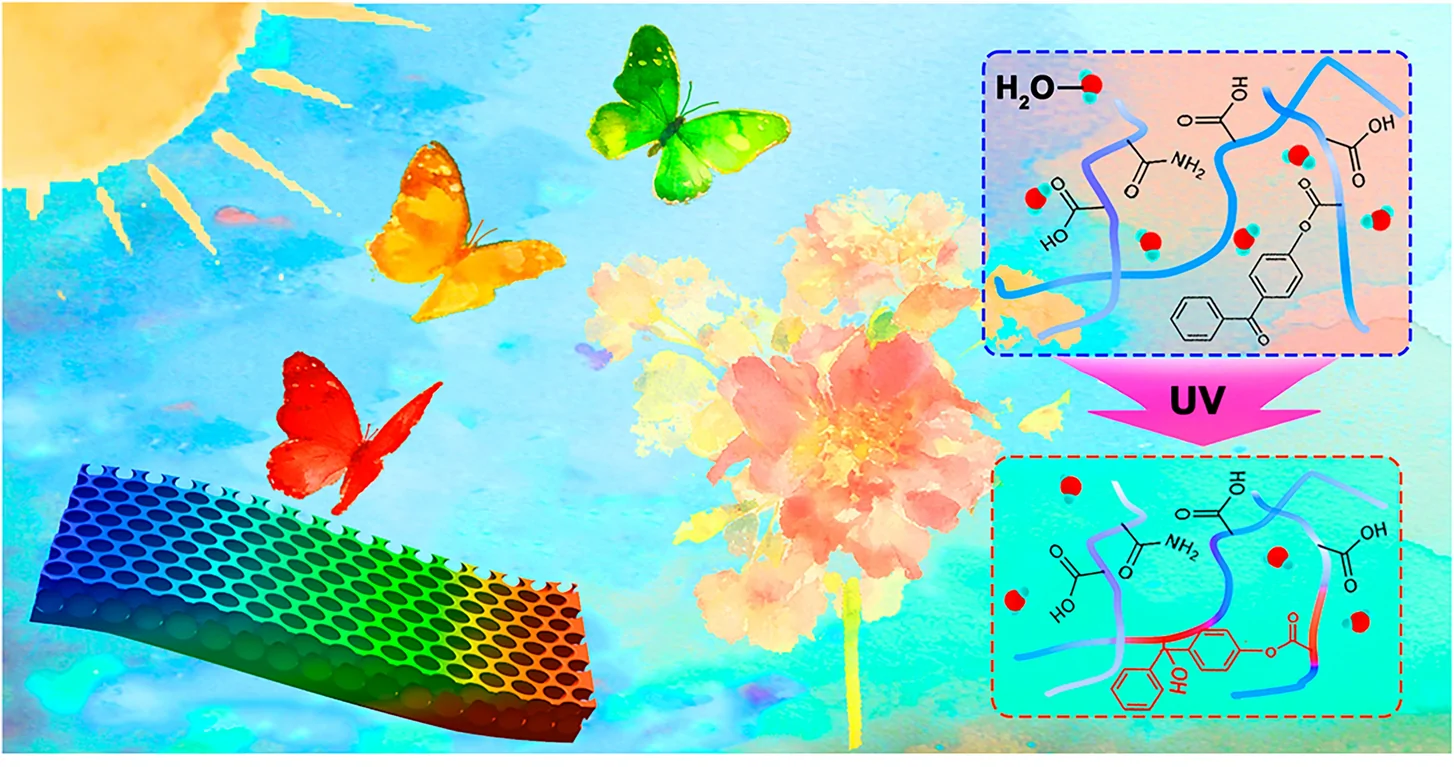

## Introduction

Information security is crucial for protecting national security, economic stability, and personal privacy [1–3]. Traditionally, techniques such as information encryption, anti-counterfeiting, and traceability have relied on complex algorithms or specialized chemical materials. However, these conventional methods are increasingly facing challenges such as computational power attacks and counterfeit reproduction [4–6]. Therefore, there is an urgent need for innovative information security technologies that are resistant to both imitation and cracking, even with the current computing power available.

In response, structural color technology has emerged as a promising solution in the field of information security [7–10]. Unlike traditional color generation, which relies on chemical pigments, structural colors are created through the interaction of light with periodic micro- and nanostructures at specific wavelengths [11, 12]. This unique physical mechanism provides features such as high resolution [13, 14], iridescence [15, 16], and environmental sustainability [17]. More importantly, by manipulating microstructural parameters, such as the lattice constant in opal photonic crystals, structural colors can be made to dynamically change in response to external stimuli [18–22]. This capability mimics the intelligent color shifts seen in chameleon skin [23, 24], offering a platform for next-generation optical anti-counterfeiting labels and information encryption technologies.

However, to effectively utilize structural colors in information security, it is essential to develop techniques for patterning them. A single, uniform structural color carries limited information, making it challenging to encode complex data or create identifiable patterns. Currently, the primary method for producing stimulus-responsive structural color patterns relies on laser etching templates [25–28]. Although this technique can achieve precise patterns, it requires costly laser systems, making it expensive. Furthermore, since structural colors depend on selective light reflection from periodic structures, the resulting patterns typically exhibit only one color, and secondary coloring or post-processing modifications are difficult to implement. These limitations hinder the broader application of this technology in practical scenarios.

To address this, many researchers have proposed their own solutions. For instance, Yu et al. [29] utilized the excellent adhesion property of polymers to cut and reattach different colored structural color gels, thereby fabricating multi-color patterned films; while Sun et al. [30] utilized light-stimulated-responsive complex fluorescent molecules to achieve dual-mode patterned display of liquid crystals and fluorescence; Shen et al. [31] used light-stimulated-responsive anthracene derivatives to prepare protein stone gel-like structural colors, and realized anti-counterfeiting applications of stretch pattern display.

**Scheme 1:**
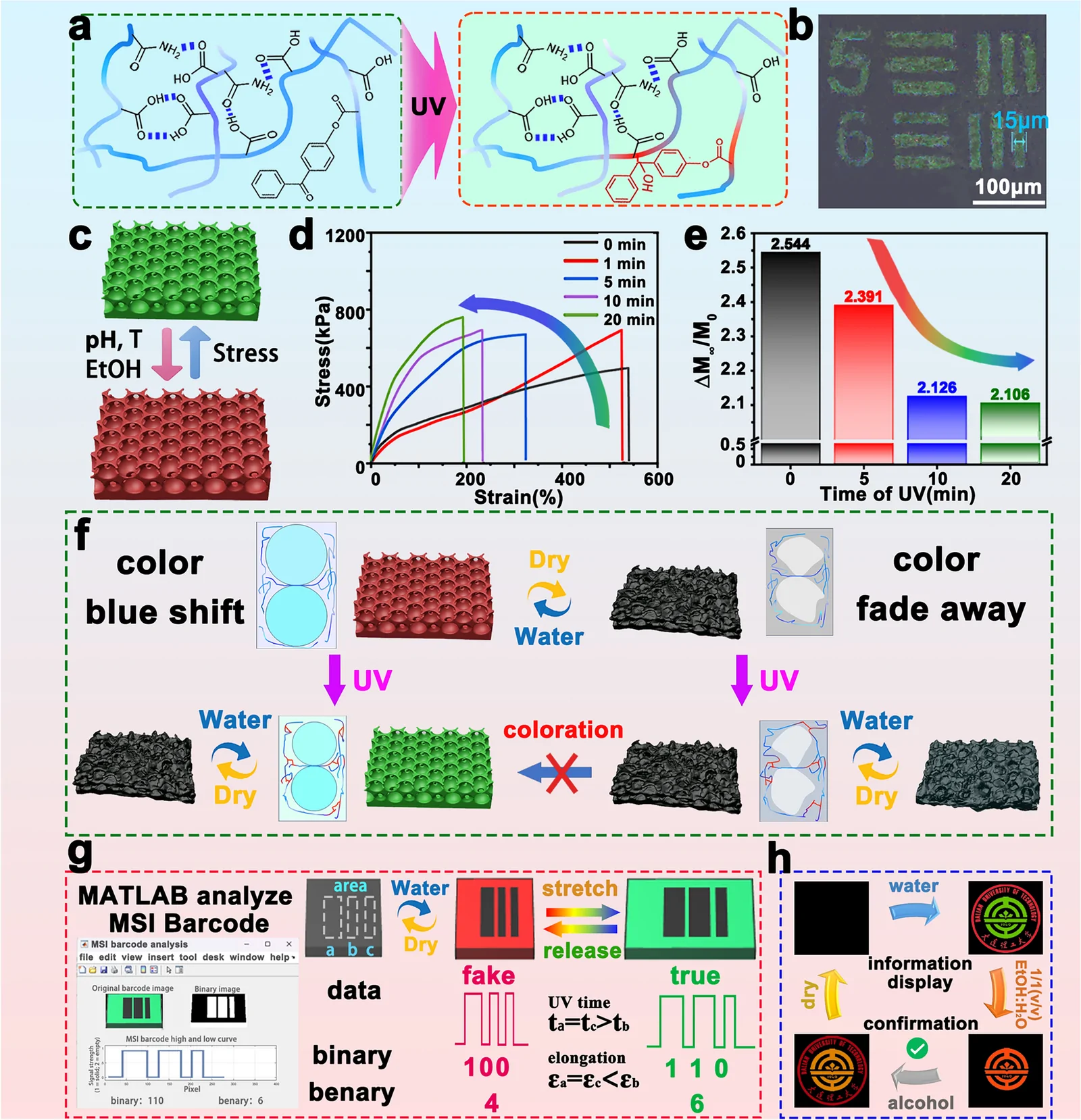
Schematic diagram of PAMBP photo-patterning and anti-counterfeiting application. **a** Schematic diagram of possible molecular chains of PAMBP.** b** Optical photograph of patterned PAMBP. **c** Stimulus response behaviors of PAMBP. **d** Changes in mechanical properties and **e** swelling capacity of PAMBP before and after photo-induced crosslinking. **f** Schematic diagrams of photo-patterning. **g**, **h** Schematic diagrams of PAMBP application in information security

Here, we propose a novel patterning strategy. First, we create structural colors across the entire film, followed by patterning to generate two or more distinct color patterns on the same film. Specifically, we introduced the photoinitiator 4-acryloyloxybenzophenone (ABP) together with acrylic acid (AA) and acrylamide (AM) into the opal template. After thermal curing and etching, we obtained an anti-opal structural color hydrogel (PAMBP). This hydrogel film displays clear structural color in its swollen state, but upon drying, the pore structure collapses and the color fades. In addition, the ABP molecules can initiate molecular chain cross-linking under ultraviolet (UV) light [32, 33], which reduces the hydrogel’s swelling capacity and alters its mechanical properties, such as tensile strength. The anti-opal hydrogel also exhibits excellent tensile properties and responds significantly to external stimuli, including stress, temperature, and solvents. These features give the structural color film unique capabilities (Scheme 1): (1) Exposure to UV light on the dry film can fix the collapsed pore structure, making the structural color disappear permanently; (2) When the film is water-soaked and exposed to UV light, the pore structure remains, but the increased cross-linking weakens the swelling ability, causing the pores to contract and resulting in a blue shift in the structural color; (3) In UV-exposed areas, the increased cross-linking and reduced swelling capacity cause the material to exhibit a lower strain under the same stress. With these photo-patterning characteristics and the film’s inherent response behavior, it is possible to create multi-region and multi-color patterns through spatially controllable optical cross-linking. This strategy offers a novel approach for high-security, hard-to-imitate information encryption and anti-counterfeiting applications.

## Experimental Section

### Preparation of SiO_2_ Nanospheres

Monodispersed SiO_2_ nanospheres were prepared by the modified Stöber method. Ethanol (200 mL) was added to ammonia solution (100 mL) with different concentrations, and a mixed solution of tetraethyl orthosilicate (40 mL) and ethanol (160 mL) was then added under magnetic stirring (1500 rpm min^−1^) at room temperature. After 2 min, the rotating speed was reduced to 600 rpm min^−1^, and the reaction was continuously stirred for 5 h to obtain the white emulsion of SiO_2_ nanospheres. The particle sizes of SiO_2_ nanospheres were regulated by varying the volume of the seed solution.

### General Procedure of Hydrogel Photonic Films

The hydrophobic structural color films were fabricated using a sacrificial template method. First, the monodispersed SiO_2_ nanospheres were synthesized, then washed with water and ethanol, and an ethanol dispersion of 8 wt% was prepared for further use. Next, the SiO_2_ colloidal crystal templates were prepared by the dip-coating method. During the ethanol volatilization, SiO_2_ nanospheres were self-assembled on the glass substrates.

Then, the prepared mixed solution of AA-AM-ABP monomers (1 mL) (quality score of AA: AM: ABP:BMAA:AIBN is 3:1:1.8:0.05:0.05) was infiltrated into the space among the SiO_2_ nanospheres of the templates under vacuum and capillary force. Following the thermal polymerization, the SiO_2_ templates were immersed in 4 vol% hydrofluoric acid and finally rinsed with deionized water, forming the hydrogel photonic films (PAMBP).

### General Procedure of PAMBP Patterning

The preparation method of PAMBP with the USAF 1951 resolution test card patterns of the United States Air Force: Dry the PAMBP hydrogel, cover it with a USAF 1951 resolution test card mask of the United States Air Force, expose it to UV light, and then immerse it in water. Through the above methods, a USAF 1951 resolution test card patterned PAMBP film can be obtained.

The heterologous butterfly pattern was prepared as follows: the preparation method of the heterologous butterfly pattern: First, under dry conditions, place the butterfly-shaped mask on the PAMBP (where the reverse opal structure collapses, so the structural color does not appear). Expose the film to 365 nm ultraviolet light (1 W cm^−2^) for 5 min. Then, immerse the PAMBP film in water, and the butterfly pattern appears quickly. Cover half of the butterfly area with the mask. PAMBP films in water were irradiated with 365 nm ultraviolet light for 20 min. The ultraviolet light and the mask were removed. It was found that the color of the irradiated area shifted blue, and a PAMBP patterned with heterochronic butterflies was obtained. Other patterning methods are similar.

## Results and Discussion

### Design and Preparation of Optical Patterned Structural Color Films

**Fig. 1:**
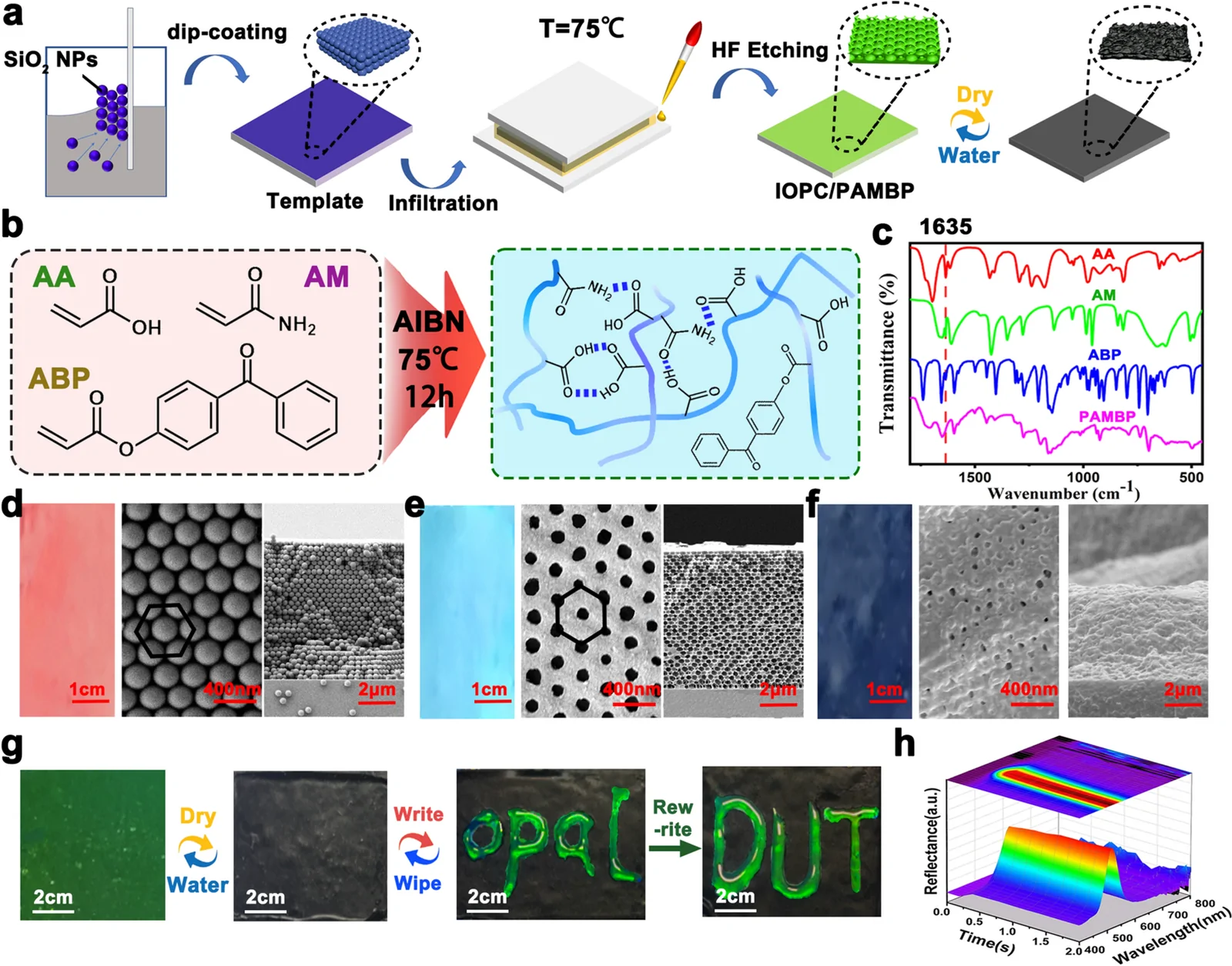
Preparation and characterization diagram of PAMBP. **a** Schematic diagram of PAMBP preparation and water response. **b** Schematic diagram of monomers for PAMBP preparation, possible copolymer chains and interchain hydrogen bonds. **c** Fourier infrared absorption spectra of monomers and PAMBP. **d-f** Optical images, surface SEM images and cross-sectional SEM images of opal templates, freeze-dried PAMBP and volatilized dried PAMBP. **g** Optical images of PAMBP water response and water writing paper application. **h** Time-resolved reflection spectra of PAMBP water response

The process for preparing photocross-linkable and patternable structurally colored films is outlined in Fig. 1a, with the corresponding reaction shown in Fig. 1b. First, SiO₂ nanospheres of varying particle sizes are synthesized using the Stöber method (size parameters are detailed in Fig. S1 and Table S1). These nanospheres are then assembled into opal templates exhibiting structural color through the impregnation and lifting method (Fig. S2). As the particle size of the SiO₂ nanospheres increases, the structural color of the template shifts toward longer wavelengths, or a redshift, in accordance with Bragg diffraction theory (Eq. S1) [15, 23, 34]. For example, the scanning electron microscopy (SEM) image (Fig. 1d) of the template assembled with 354 nm SiO₂ nanospheres shows a face-centered cubic (FCC) structure [35–37].

To prepare the hydrogel film, we dissolved the copolymer monomer acrylamide (AM), photo-crosslinking molecule ABP, thermal initiator azodiisobutyronitrile (AIBN), and crosslinking agent N,N-Methyldipropylenecarboxamide (MBAA) in acrylic acid (AA) to create the precursor solution. This solution was injected into the template, and after heating and curing, the SiO₂ nanospheres were etched away using an HF solution. The residual HF was replaced with deionized water and ethanol. Finally, after soaking in deionized water, a series of anti-opal hydrogels with different structural colors (referred to as PAMBP, with color changes shown in Fig. S2) were successfully obtained. As the particle size of the template nanospheres increased, the structural color of the hydrogel also exhibited a redshift, consistent with the Bragg diffraction law.

We conducted infrared spectroscopy analysis on the resulting PAMBP hydrogel (Fig. 1c), and found that the stretching vibration peak of the carbon–carbon double bond at 1635 cm⁻^1^ disappeared completely, confirming that the polymerization reaction had been fully completed. In addition, energy-dispersive spectroscopy (EDS) analysis (Fig. S3) revealed that the characteristic Si peaks in the etched samples were absent, indicating complete removal of the SiO₂ template.

SEM images of the freeze-dried PAMBP (Figs. 1e and S4) show a well-defined pore structure, successfully replicating the FCC structure of the opal template. Because we used a sandwich structure to inject the precursor solution to initiate the polymerization, and then further etched to obtain the PAMBP structured color film, the anti-opal structure is located on the surface of the PAMBP film, and its thickness is only a few nanometers (Fig. S4). Different thicknesses of anti-opal structures can be obtained by changing the number of coating and pulling times (Fig. S5). Increasing the number of coating and pulling times can enhance the thickness of the anti-opal structure, make the color more vivid, and increase the maximum reflectance corresponding to the reflection spectrum (Fig. S6). When the number of coating and pulling times reaches more than 6, the thickness of the anti-opal structure reaches 5.7 μm. When the number of coating and pulling times was further increased, the structural color change in PAMBP was not obvious, while the time required for preparation, SiO_2_ nanospheres, and HF acid all increased. Therefore, the template of impregnation and lifting six times was adopted in the subsequent experiments.

The pore size is smaller than that of the template nanospheres, due to the hydrogel’s shrinkage during freeze-drying. When the hydrogel is air-dried, the pore structure collapses (Fig. 1f), causing the structural color to disappear. The PAMBP film exhibits excellent stability. By testing its reflection spectra during the drying-water soaking cycle (Fig. S7), it can be observed that after 100 cycles, the structural color remains almost unchanged, indicating its outstanding water-responsive stability. This feature enables applications like water-rewritten paper (Fig. 1g) and water-activated anti-counterfeiting (Fig. S8), with a water response time of less than 0.5 s (Fig. 1h and Video S1).

**Fig. 2:**
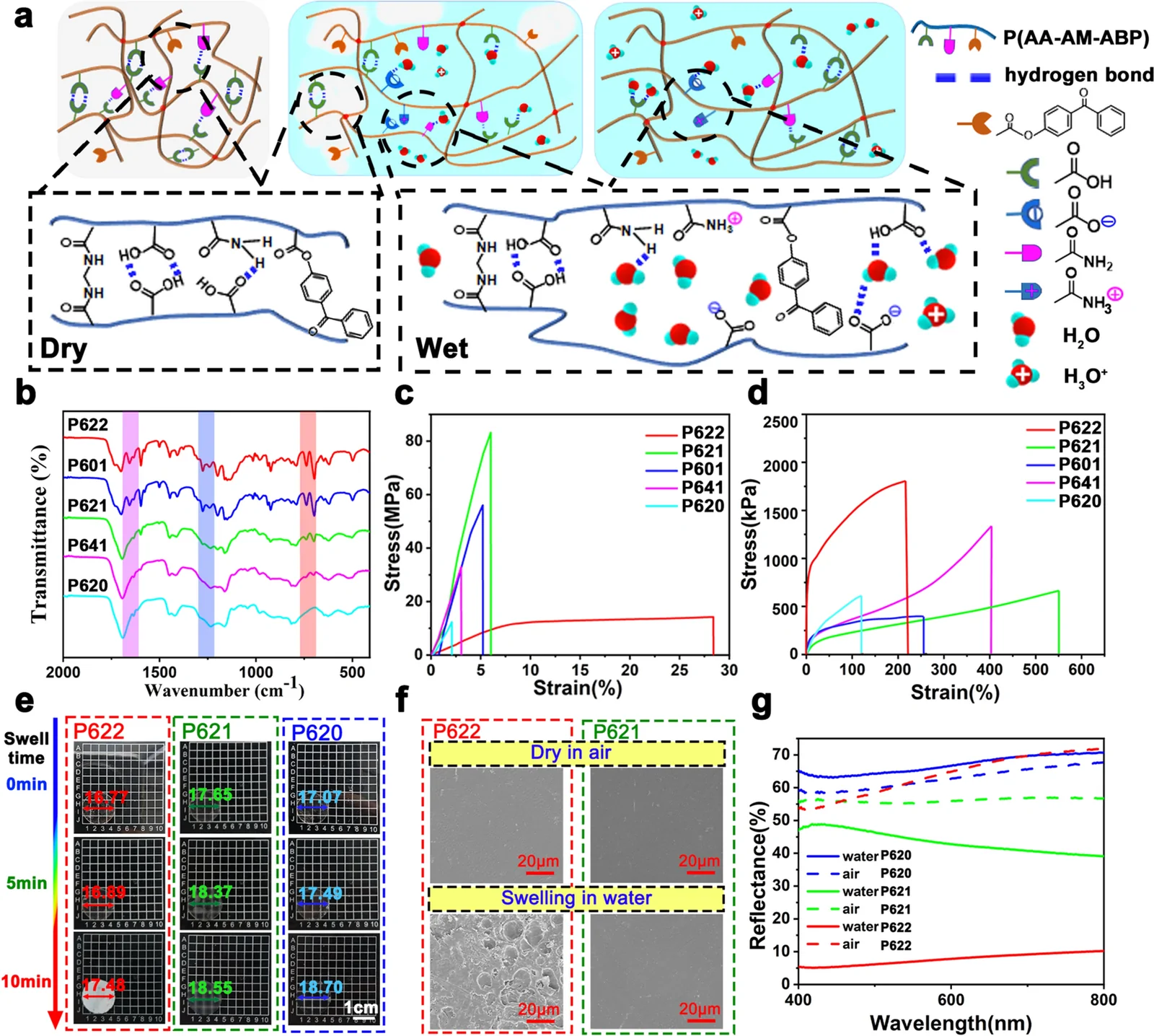
Performance characterization of PAMBP with different raw material ratios. **a** Schematic diagrams of possible polymer chains and interchain hydrogen bonds of hydrogels in dry, semi-wet and wet states. **b-d** FT-IR spectra of P622, P601, P621, P641, and P620, as well as the tensile fracture curves in dry and wet states. **e** Optical images of P622, P621, and P620 swelling in deionized water at 0, 5, and 10 min, respectively. **f** Cross-sectional SEM images of P622 and P621 in a dry state and after being thoroughly soaked in deionized water and freeze-dried. **g** Transmission spectra of P622, P621, and P620 under dry conditions and after being thoroughly soaked in deionized water

To study the effect of ABP on the performance of the structural color films, we first optimized the PAMBP raw material ratio. We prepared five PAMBP hydrogel films with molar ratios of AA, AM, and ABP of 6:2:2, 6:2:1, 6:2:0, 6:0:1, and 6:4:1, which were named P622, P621, P620, P601, and P641, respectively. Infrared spectroscopy analysis (Fig. 2b) revealed that as the ABP content increased, the absorption peaks corresponding to the C-H out-of-plane bending vibration of the benzene ring at 750 cm⁻^1^ and the stretching vibration of the benzene ring skeleton at 1600 cm⁻^1^ became more pronounced. Additionally, when the AM content increased, the absorption peak near 1250 cm⁻^1^, corresponding to the coupling mode of C-N stretching and N–H bending vibrations in the primary amide, also intensified.

Tensile performance tests were conducted on the hydrogels mentioned above (Fig. 2c, d). The results show that the hydrogel’s elongation at break significantly increased after 12 h of water immersion. Notably, the fracture strain of P621 exceeded 500% (Fig. S9), although its fracture stress decreased. This behavior can be attributed to the effect of water molecules on the dynamic properties of the polymer network, enhancing energy dissipation, preventing crack propagation, and improving network ductility. In the dry state, the molecular chains are closer together and form interchain entanglements, reducing the material’s toughness [38, 39]. Interestingly, the P621 hydrogel demonstrated excellent ductility while maintaining some tensile strength: a P621 sample with a cross-sectional size of 0.3 mm × 2 mm was able to lift a 100 g weight (Fig. S5) and achieve a tensile strain of up to 500%.

In addition, after full water immersion, the P622 sample show noticeable whitening, while the light transmittance of P621 and P620 remained relatively stable (Figs. 2e, g and S10). This difference can be explained by ABP’s hydrophobic nature, which reduces the overall hydrophilicity of the film. In P622, the high ABP content triggered microphase separation during water absorption, creating an inhomogeneous structure with dry and swollen regions coexisting [32, 40] (Fig. 2a). This phase separation caused local differences in the refractive index, leading to strong light scattering and the whitening effect. In contrast, P621 and P620, which had lower ABP content, exhibited more uniform water absorption with no significant phase separation. SEM analysis of P622 and P621 in both dry and wet freeze-dried states (Figs. 2f and S10) show micron-sized pores in P622, further confirming the microphase separation structure. The structure of P621 was uniform with no such phenomenon, consistent with the proposed mechanism. In conclusion, P621 has good light transmission performance, does not affect the color of the anti-protein structural color, and has good mechanical properties. Therefore, this study identifies P621 as the optimal polymer substrate for constructing anti-opal structurally identifies hydrogels. All subsequent experiments were conducted using the PAMBP material prepared according to this formulation.

### Response Behavior of PAMBP

**Fig. 3:**
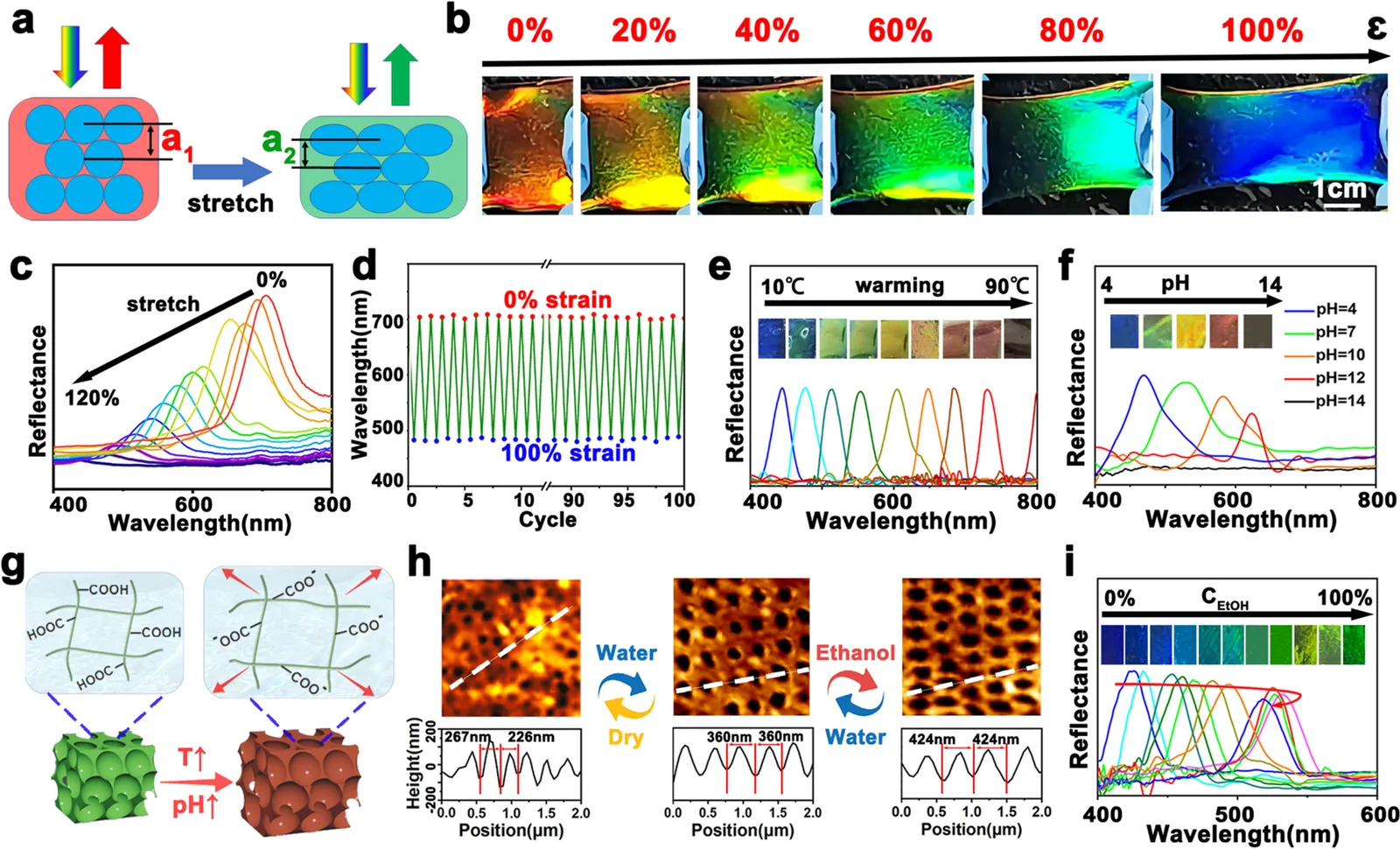
Relevant characterization of PAMBP stimulus response behavior. **a** Schematic diagrams of pore structure and structural color changes in stretched PAMBP. **b**, **c** PAMBP tensile color-changing optical photograph and reflection spectra. **d** Changes in reflection wavelength during 100 load-unloading cycles. **e**,** f**, **i** Reflection spectra and optical images of PAMBP impregnated with solvents of different temperatures, pH values and ethanol concentrations. **g** Schematic diagram of the redshift of PAMBP structural color. **h** Atomic force microscope image of the surface of PAMBP impregnated with different

The AM-AA system responds to various stimuli. When combined with structural colors, it will produce wonderful phenomena [41–43]. Research on the response behavior of PAMBP structural color films reveals that the material can respond to a range of external stimuli, including stress, temperature, pH, and alcohol-water solvents. When the PAMBP anti-opal structural color hydrogel film is stretched, the effective lattice constant of its anti-opal structure decreases (Fig. 3a). According to Bragg’s diffraction law (Eq. S1), this reduction in lattice constant results in a blue shift of the structural color. Reflection spectra measured during the tensile process (Fig. 3c) show a noticeable blue shift as the film deforms (Fig. 3b and Video S2). The mechanical chromic sensitivity of the material is impressive, reaching 2.5nm per 1% distortion with a maximum wavelength shift (Δλ) exceeding 260 nm. This excellent response performance is attributed to the hydrogel’s high stretchability and the periodic structure of the anti-opal film. A loading–unloading cycle test under 100% strain (Fig. 3d) revealed no significant drift in the optical signals after 100 cycles, indicating strong cycle stability.

The PAMBP film also demonstrates a sensitivity to temperature. This system contains a large number of carboxyl groups and hydrogen bonds within the polymer chains. As the temperature rises, the dissociation of these carboxyl groups becomes more intense, and the electrostatic repulsion between molecular chains increases [44, 45]. Moreover, as the temperature rises, the hydrogen bonds within the polymer break [46, 47]. These two factors jointly lead to the expansion of the hydrogel (Figs. 3g and S12a). By taking photographs of circular PAMBP films with swelling equilibrium at different temperatures (Fig. S11) and measuring the film radius, it can be found that as the temperature rises, the degree of film swelling increases. Meanwhile, variable-temperature infrared spectroscopy also demonstrated that as the temperature rose, the water content within the hydrogel increased (Fig. S12b). Moreover, through pH meter measurement, it was found that the pH decreased significantly (Fig. S12c, d), which proved that the degree of dissociation did indeed increase with the rise in temperature. As the temperature increases, the swelling of PAMBP results in a red shift of its structural color (Fig. 3e). Moreover, after 100 cycles from 20 to 60 °C H_2_O soaking, the response characteristics of PAMBP to temperature remained almost unchanged, indicating that it has good cycling stability (Fig. S13). In addition, when an infrared laser is directed at PAMBP samples doped with carbon black, the photothermal conversion of carbon black raises the local temperature, inducing a red shift in the structural color of the irradiated area (Fig. S14).

The PAMBP film also demonstrates a sensitivity to pH. The system contains a large number of carboxyl groups, which dissociate more intensely as pH increases. This increased dissociation leads to greater electrostatic repulsion between the carboxylate ions, causing the hydrogel to swell (Fig. 3g) [48, 49]. By taking photographs of circular PAMBP films with swelling equilibrium at different pH values (Fig. S15) and measuring the film radius, it can be found that as the pH increases, the degree of film swelling increases. When the pH rises to 14, the film radius increases significantly and wrinkles appear. The films also exhibit a pH response. As the pH increases from 4 to 12, the reflection peak shifts from 460 to 630 nm, indicating a red shift in the structural color. Moreover, after 100 solvent immersion cycles with pH values of 4 and 12, the response characteristics of PAMBP to pH remained basically unchanged, indicating that it has good cycling stability (Fig. S16). When the pH reaches 14, the structural color disappears entirely (Fig. 3f). This is because the film's swelling capacity has increased too much, and its color has redshifted beyond the visible light region.

Furthermore, PAMBP films respond significantly to alcohol-water solvents. As the ethanol concentration increases, the reflection wavelength initially shifts to the red and then shifts back to the blue (Fig. 3i). Moreover, after 100 cycles of H_2_O-EtOH soaking, the response characteristics of PAMBP to alcohol-water solvents remained basically unchanged, indicating that it has good cycling stability (Fig. S18). This behavior can be explained as follows: PAMBP, being a polymer hydrogel, is highly compatible with ethanol. As the ethanol concentration increases, the hydrogel swells, causing a red shift in its structural color. However, as the ethanol concentration continues to rise, the dissociation of carboxyl groups decreases, electrostatic repulsion weakens, and the swelling reduces, causing a blue shift in the structural color. By photographing circular PAMBP films with swelling equilibrium at different ethanol concentrations (Fig. S17) and measuring the film radius, it can be found that as the ethanol content increases, the degree of film swelling first increases and then decreases, verifying the above response mechanism. This mechanism is further supported by atomic force microscopy (AFM) images (Fig. 3h), which show that the pore structure partially collapses in the dry state, swells in water, and shows a visible structural color. Upon exposure to ethanol, the pore spacing increases from 360 to 424 nm, resulting in a red shift of the structural color.

Building on the optical response characteristics of PAMBP to solvent stimuli, we developed a chrysanthemum-patterned sensing array (Figs. S19 and S20) for detecting ethanol–water solutions. This array can output matrix optical signals, which not only enhance detection accuracy but also improvethe fault tolerance of the system.

## Research on the Photo-Initiated Crosslinking Process

**Fig. 4:**
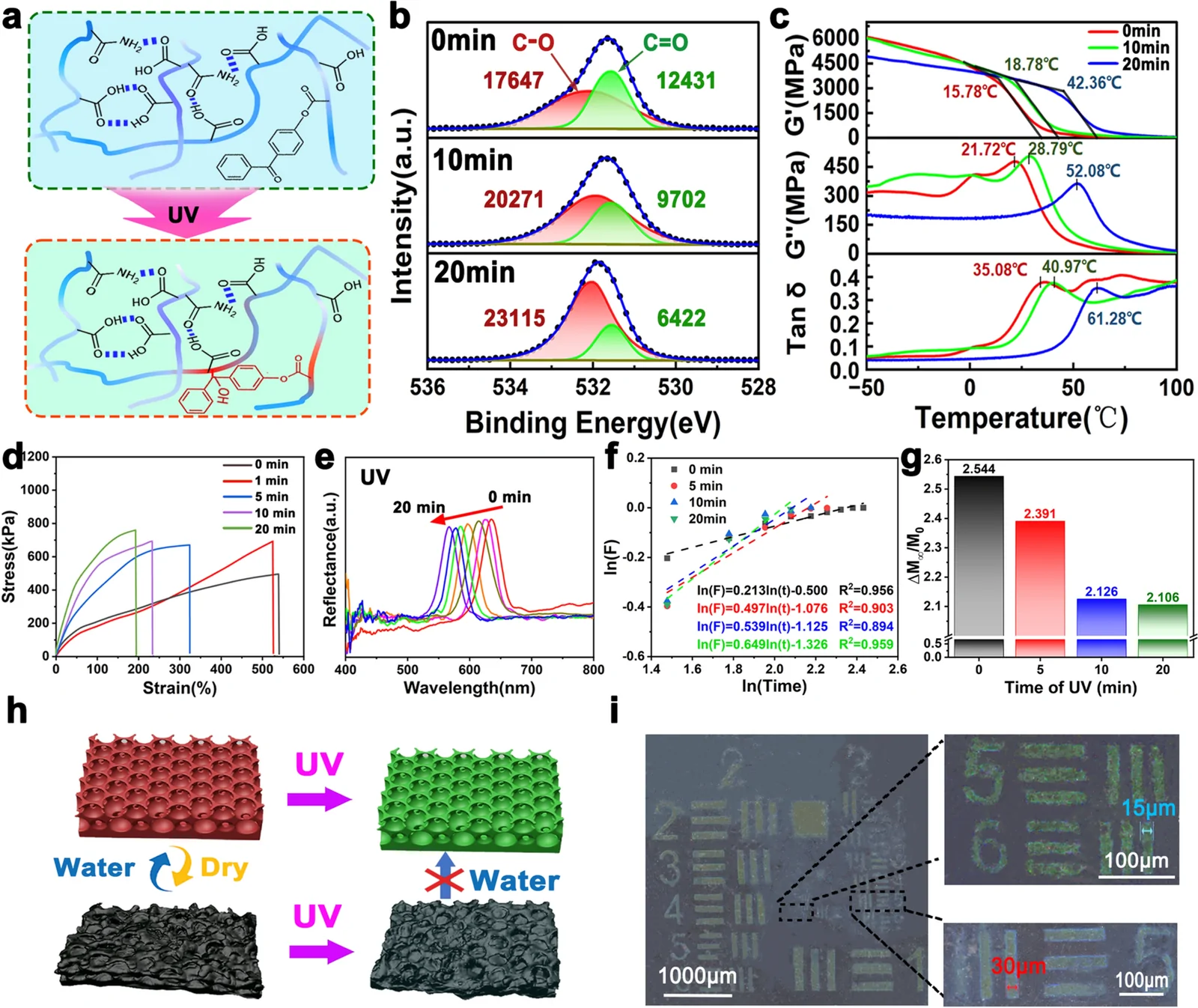
Relevant representations of PAMBP photo-patterning behavior.** a** Schematic diagrams of possible copolymer chains and inter-chain hydrogen bonds of PAMBP, as well as possible copolymer chains and inter-chain hydrogen bonds after photo-crosslinking. **b** O-1*s* peak fitting spectra of XPS, **c** DMA diagrams, **d** stress–strain curves, **e** reflection spectra, **f** swelling kinetics curves and **g** swelling equilibrium mass ratio bar charts at different times of PAMBP photo-crosslinking. **h** Schematic diagram of PAMBP patterning.** i** Ultra-depth-of-field microscope images of PAMBP patterned by the USAF 1951 resolution test card mask of the United States Air Force

The crosslinking process of PAMBP hydrogel films under UV light irradiation was deeply studied. Previous studies, such as those by Liu et al., have shown that UV light can excite the benzophenone group, initiating a hydrogen extraction reaction [32, 50]. This reaction generates a benzyl alcohol structure and forms new C–C bonds, which increases the crosslinking density of the film (Fig. 4a). We verified this mechanism through XPS and DMA tests. The XPS peak fitting results for the O 1*s* orbital of the hydrogel film after 0, 10, and 20 min of UV irradiation are shown in Fig. 4b. As the irradiation time increases, the peak corresponding to the C = O group gradually decreases, while the peak for the C–O group increases, confirming the transformation from benzophenone to benzyl alcohol. DMA results (Fig. 4c) also show that as UV irradiation time increases, the glass transition temperature (T_g_) of the hydrogel rises, together with the maximum loss modulus and the temperature corresponding to the maximum tanδ. These changes indicate a significant increase in the crosslinking degree of the material as the UV light reaction progresses [51]. Therefore, the tensile stress of the material increases with the higher crosslinking degree, while the fracture strain decreases (Fig. 4d). Thermal gravimetric analysis (TGA) (Fig. S21) shows a slight increase in the thermal decomposition temperature with longer UV exposure.

To visually demonstrate the effect of UV light on the crosslinking degree, we used a mold press to shape the hydrogel films into a “flower” shape (Fig. S22). When placed in room-temperature water and exposed to UV light on the top surface, the “flower” gradually closed as the exposure time increased (Video S3). This phenomenon can be explained by UV light predominantly affecting the top surface of the film, leading to a higher crosslinking density there. This causes the bottom surface to retain a greater swelling capacity in water (Fig. S22a). The asymmetry in swelling behavior causes the “flower” structure to bend inward, eventually closing up.

To confirm this mechanism, we conducted swelling tests on hydrogel films exposed to uniform UV light on both surfaces for different durations. Figure S23 shows the scatter plot of the hydrogel’s swelling mass over time after exposure to UV light for 0, 5, 10, and 20 min. By fitting the experimental data to the Equation in Formula S2, we obtained the ln(F)—ln(t) relationship curve (Fig. 4f). The results show that as UV exposure time increases, the fitting coefficient (a) rises from 0.213 to 0.649, indicating that the swelling behavior shifts from the Fick diffusion model (a < 0.5) to a non-Fick diffusion model (0.5 < a < 1) [52, 53]. Without UV treatment, the swelling follows the Fick diffusion model, but after UV exposure, the relaxation of macromolecular chains significantly affects the water absorption process. In addition, as UV exposure time increases, the volume change of the hydrogel at equilibrium swelling decreases (Fig. 4g), and the water absorption mass ratio (Δm/m₀) drops from 2.544 to 2.106. This suggests that increased crosslinking effectively reduces the swelling capacity of the material. The reduced swelling capacity causes the pores in the opal structural color hydrogel film to shrink, which results in a blue shift of the structural color (Fig. 4e).

Based on the photocuring crosslinking characteristics of PAMBP structural color films, we developed two photocuring procedures for patterning the films (Fig. 4h). The first uses photocuring in water, which induces a blue shift in the structural color, while the second occurs under dry conditions, causing the structural color to disappear. We also explored the ultimate resolution of this patterning method. Using the USAF 1951 resolution test card as the mask (Fig. 4i), we determined that the minimum line width achievable is 15 μm, demonstrating the high resolution of this patterning technique for structural color films.

The high-resolution patterning of this film is formed by the light-induced cross-linking locking of the collapsed pore structure, which cannot restore the reverse opal structure. Since the patterning originates from the anti-opal structure on the upper layer of the film, the thickness of the film has almost no effect on the patterning resolution, while the thickness of the anti-opal layer may have a certain impact on the patterning resolution. Therefore, we supplemented the pattering experiments of PAMBP obtained from templates with different coating and pulling times (Fig. S24), and found that as the thickness of the anti-opal layer increased, the minimum line width of the pattering decreased, indicating that the resolution of the pattering improves when the thickness of the anti-opal layer increase. We analyzed that it might be due to the light leakage problem during the photo-patterning process. This can be confirmed by the SEM images of the cross-section and surface (Fig. S25). It can be found that after photo-patterning, the originally regular FCC structure in the masked area has also undergone a certain degree of distortion and deformation. It is precisely the slight light leakage of the mask that leads to the decrease in resolution during the PAMBP photo-pattering process. Due to the limited penetrating power of light, when the number of layers of opal is small, the impact of light leakage is greater.

### Anti-Counterfeiting Application of Structured Color Films

**Fig. 5:**
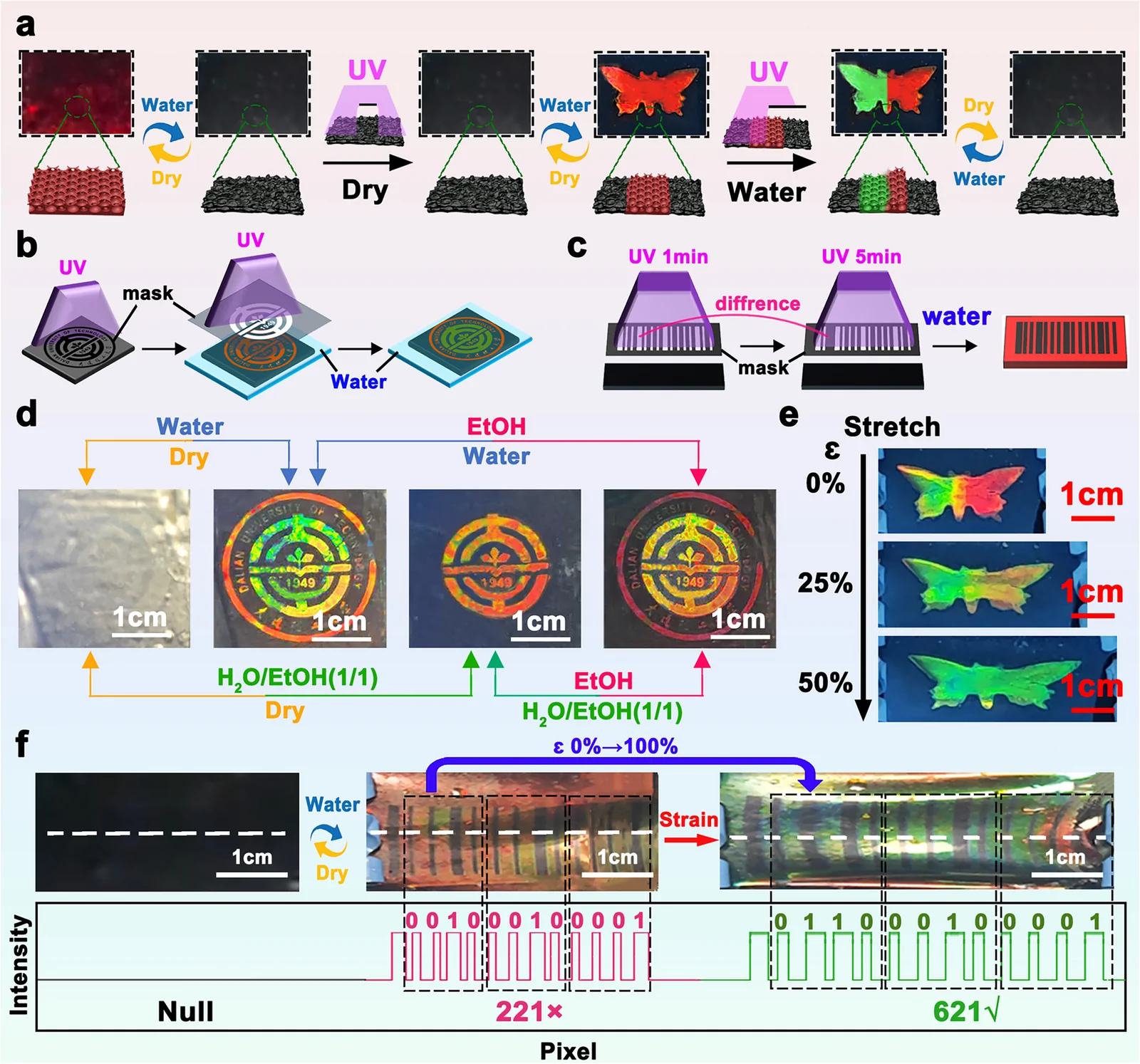
Relevant characterization of PAMBP application in information security examples. Schematic diagram of PAMBP preparation with** a** dual-color butterfly, **b** Dalian University of Technology emblem and** c** MSI code pattern. Anti-counterfeiting mechanism and optical photographs of PAMBP with **d** Dalian University of Technology emblem,** e** dual-color butterfly, and **f** MSI code pattern

By combining the photo-induced crosslinking patterning property of PAMBP with its stimulus–response characteristics, we have developed a structured color film with both information encryption and anti-counterfeiting capabilities. To demonstrate this, we first prepared a red and green dual-color butterfly pattern film, and the preparation process is illustrated in Fig. 5a. Initially, the dry PAMBP film is exposed to UV light through a mask to fix the collapsed pores in the areas outside the butterfly pattern. This allows a red butterfly pattern to appear when the film was immersed in water. Then, the left half of the butterfly pattern is exposed to UV light while the film was in water. The increased crosslinking in this region reduces its swelling capacity, causing a blue shift in its structural color. This process results in a red and green butterfly pattern. The anti-counterfeiting feature of this film is evident in several ways: in its dry state, it is colorless, but when it comes into contact with water, the butterfly pattern becomes visible. Additionally, when stretched laterally, the green area, with its higher degree of crosslinking, has a lower tensile elongation, while the red area deforms more easily, showing greater elongation. This mechanical response can further serve as an anti-counterfeiting verification method (Fig. 5e and Video S4).

Building on the characteristic solvent-responsive behavior of PAMBP (Fig. 3i), we also developed a new solvent-responsive anti-counterfeiting mechanism (Fig. 5d). The red and green dual-color structural color films were designed using the emblem pattern of Dalian University of Technology (Fig. 5b). The central area of the emblem, which was subjected to a higher degree of UV crosslinking, has a lower swelling capacity compared to the peripheral area. In its dry state, the film is colorless, but when exposed to water, the outer pattern turns red and the central emblem turns green. When placed in a 1:1 alcohol-water solution, the outer pattern shifts to the infrared spectrum, becoming invisible to the human eye, while the central pattern turns red. In pure ethanol, the outer pattern appears deep red, while the central emblem turns yellow. This multi-solvent color response behavior makes the film ideal for advanced information security and anti-counterfeiting applications, offering multiple layers of verification.

Lastly, we leveraged the effect of crosslinking on the material’s stress–strain behavior to create PAMBP films with MSI barcode patterns (Fig. 5c). In its dry state, this film does not display any information. However, after soaking in water, it shows false information, and only when stretched does it reveal the true data (Fig. 5f). To decode the pattern, we used MATLAB to write a pattern recognition script, which converted the MSI barcode pattern into a fluctuating curve, enabling us to analyze its binary data. Specifically, the corresponding MATLAB program script first reads the MSI optical image, then performs grayscale processing and binarization analysis to obtain a black-and-white image. It further extracts binary data, finally converts it into decimal values, and outputs the obtained data (Fig. S26). The areas exposed to UV light for a longer period (except for the third barcode) exhibited less deformation during stretching, resulting in an initial recognition of "221." After stretching, the third barcode area, which had less crosslinking, deformed significantly, changing the final recognition result to "621." This dynamic, mechanically triggered information display provides a new strategy for information security and encryption.

## Conclusion

This study presents a novel approach to preparing structural color films using a “film formation first, then patterning” strategy. We successfully created anti-opal hydrogel structural color films that respond to various stimuli, including stress, temperature, and solvents. These films are transparent and colorless in their dry state but exhibit structural color upon contact with water. By introducing the photo-crosslinking molecule ABP, we achieved patterned regulation through UV irradiation: in the dry state, UV exposure causes the structural color in the irradiated area to disappear, while exposure in water induces a blue shift in the color. Through light-induced, regionally differentiated crosslinking, we were able to spatially regulate the film’s mechanical properties and swelling behavior, resulting in patterned structural colors. This method enables the creation of patterns with a line width as small as 15 μm, greatly enhancing the resolution of structural color films. By combining these responsive characteristics, we successfully created both multi-color and single-color patterned films, and demonstrated three applications suitable for information encryption and anti-counterfeiting. This research offers new approaches for developing intelligent anti-counterfeiting and encryption technologies and highlights the broad potential of responsive sensing applications.

## Supplementary Information

Below is the link to the electronic supplementary material.

Supplementary file1 (DOCX 11103 KB)

Supplementary file2 (MP4 2292 KB)

Supplementary file3 (MP4 1073 KB)

Supplementary file4 (MP4 1255 KB)

Supplementary file5 (MP4 1847 KB)

## Abbreviations

s: Seconds
min: Minutes
g: Grams
h: Hours
mL: Milliliters
mm: Millimeter
nm: Nanometer
μm: Micrometer
m: Mass(g)
T: Temperature (°C)
t: Time (min)
r min^−^^1^: Rotate per minute
wt%: Mass fraction percent
vol%: Volume fraction percent
W cm^−^^2^: Watt per square centimeter

## List of symbols

a: Fitting coefficient
AA: Acrylic acid
ABP: 4-Acryloyloxybenzophenone
AIBN: Azo diisobutyronitrile
AM: Acrylamide
F: Water retention rates
FCC: Face-centered cubic
HF: Hydrofluoric acid
MBAA: N, N-methylene acrylamide
PAMBP: The copolymer of AA, AM and ABP
Tg: Glass transition temperature
UV: Ultraviolet

## Subscripts

M_∞_: Final mass
M_0_: Initial mass

## Greek symbols

λ: Maximum reflection wavelength
∆λ: Maximum reflection wavelength displacement

## References

[1] E. Prime, D. Solomon, Australia’s plastic banknotes: fighting counterfeit currency. Angew. Chem. Int. Ed. **49**(22), 3726–3736 (2010). 10.1002/anie.200904538 10.1002/anie.200904538 20358564

[2] Z.X. Ng, A. Ahmad, S.B. Maynard, Maynard. Information security management: factors that influence security investments in SMES. Proc. Aust. Inf. Secur. Manage. Conf. **11**, 60–74 (2014). 10.4225/75/57b56667cd8e5

[3] A.J. Palmer, Criteria to evaluate automated personal identification mechanisms. Comput. Secur. **27**(7–8), 260–284 (2008). 10.1016/j.cose.2008.07.007

[4] M. Eling, M. McShane, T. Nguyen, Cyber risk management: history and future research directions. Risk Manag. Insur. Rev. **24**(1), 93–125 (2021). 10.1111/rmir.12169

[5] L. Magnusson, S. Iqbal, P. Elm, F. Dalipi, Information security governance in the public sector: investigations, approaches, measures, and trends. Int. J. Inf. Secur. **24**(4), 177 (2025). 10.1007/s10207-025-01097-x

[6] M. Gerber, R. von Solms, Management of risk in the information age. Comput. Secur. **24**(1), 16–30 (2005). 10.1016/j.cose.2004.11.002

[7] H. Huang, H. Li, J. Yin, K. Gu, J. Guo et al., Butterfly-inspired tri-state photonic crystal composite film for multilevel information encryption and anti-counterfeiting. Adv. Mater. **35**(17), 2211117 (2023). 10.1002/adma.202211117 10.1002/adma.202211117 36739172

[8] X. Lin, Q. Li, Y. Tang, Z. Chen, R. Chen et al., Physical unclonable functions with hyperspectral imaging system for ultrafast storage and authentication enabled by random structural color domains. Adv. Sci. **11**(31), 2401983 (2024). 10.1002/advs.202401983 10.1002/advs.202401983 PMC11336904 38894574

[9] J. Zhang, C. Song, S. Zhang, S. Qin, Y. Ren et al., Time-dependent information encryption in liquid crystalline polymer with programmable glass transition temperature. Adv. Funct. Mater. **34**(28), 2400030 (2024). 10.1002/adfm.202400030

[10] J. Liu, D. Ma, C. Qi, D. Yang, S. Huang, Mechanochromic and solvomechanochromic fluorescent photonic crystals for dual-mode modulating fluorescence and multilevel anticounterfeiting. ACS Appl. Mater. Interfaces **16**(2), 2740–2750 (2024). 10.1021/acsami.3c15120 38183271 10.1021/acsami.3c15120

[11] M. Srinivasarao, Nano-optics in the biological world: beetles, butterflies, birds, and moths. Chem. Rev. **99**(7), 1935–1962 (1999). 10.1021/cr970080y 11849015 10.1021/cr970080y

[12] F. Fu, L. Shang, Z. Chen, Y. Yu, Y. Zhao, Bioinspired living structural color hydrogels. Sci. Robot. **3**(16), eaar8580 (2018). 10.1126/scirobotics.aar8580 33141750 10.1126/scirobotics.aar8580

[13] W. Li, Y. Wang, M. Li, L.P. Garbarini, F.G. Omenetto, Inkjet printing of patterned, multispectral, and biocompatible photonic crystals. Adv. Mater. **31**(36), 1901036 (2019). 10.1002/adma.201901036 10.1002/adma.201901036 31309624

[14] J. Zheng, Y. Zhang, H. Yu, J. Wang, H. Guo et al., Tunable optical metamaterial enables steganography, rewriting, and multilevel information storage. Nano-Micro Lett. **18**(1), 58 (2025). 10.1007/s40820-025-01897-9 10.1007/s40820-025-01897-9 PMC12413373 40911120

[15] W. Fan, J. Zeng, Q. Gan, D. Ji, H. Song et al., Iridescence-controlled and flexibly tunable retroreflective structural color film for smart displays. Sci. Adv. **5**(8), eaaw8755 (2019). 10.1126/sciadv.aaw8755 31448332 10.1126/sciadv.aaw8755 PMC6688865

[16] P. Xue, Y. Chen, Y. Xu, C. Valenzuela, X. Zhang et al., Bioinspired MXene-based soft actuators exhibiting angle-independent structural color. Nano-Micro Lett. **15**(1), 1 (2022). 10.1007/s40820-022-00977-4 10.1007/s40820-022-00977-4 PMC9705670 36441443

[17] X. Ma, B. Wu, L. Hou, P. Wu, Edible structurally colored plastics. ACS Nano **19**(26), 23945–23954 (2025). 10.1021/acsnano.5c05346 40561459 10.1021/acsnano.5c05346

[18] M. Li, H. Tan, L. Jia, R. Zhong, B. Peng et al., Supramolecular photonic elastomers with brilliant structural colors and broad-spectrum responsiveness. Adv. Funct. Mater. **30**(16), 2000008 (2020). 10.1002/adfm.202000008

[19] Y. Wang, M. Li, J.-K. Chang, D. Aurelio, W. Li et al., Light-activated shape morphing and light-tracking materials using biopolymer-based programmable photonic nanostructures. Nat. Commun. **12**, 1651 (2021). 10.1038/s41467-021-21764-6 33712607 10.1038/s41467-021-21764-6 PMC7955034

[20] X. Zhang, T. Yin, J. Ge, Thermochromic photonic crystal paper with integrated multilayer structure and fast thermal response: a waterproof and mechanically stable material for structural-colored thermal printing. Adv. Mater. **36**, 2309344 (2024). 10.1002/adma.202309344 10.1002/adma.202309344 37906731

[21] Y. Du, J. Zeng, Q. Sun, S. Yu, D. Yang et al., Polymerization-induced highly brilliant and color-recordable mechanochromic photonic gels for ink-free patterning. J. Colloid Interface Sci. **679**, 883–892 (2025). 10.1016/j.jcis.2024.10.178 39486227 10.1016/j.jcis.2024.10.178

[22] Y. Hu, C. Qi, D. Ma, D. Yang, S. Huang, Multicolor recordable and erasable photonic crystals based on on-off thermoswitchable mechanochromism toward inkless rewritable paper. Nat. Commun. **15**, 5643 (2024). 10.1038/s41467-024-49860-3 38969630 10.1038/s41467-024-49860-3 PMC11226673

[23] P. Vukusic, Evolutionary photonics with a twist. Science **325**(5939), 398–399 (2009). 10.1126/science.1177729 19628844 10.1126/science.1177729

[24] J. Teyssier, S.V. Saenko, D. van der Marel, M.C. Milinkovitch, Photonic crystals cause active colour change in chameleons. Nat. Commun. **6**, 6368 (2015). 10.1038/ncomms7368 25757068 10.1038/ncomms7368 PMC4366488

[25] X. Guo, R. Han, M. Kong, S. Zhang, Y. Zhang et al., Superhydrophobic and temperature-responsive fluoropolymer structural color film. Dyes Pigments **235**, 112595 (2025). 10.1016/j.dyepig.2024.112595

[26] F. Meng, Z. Wang, S. Zhang, B. Ju, B. Tang, Flexible photonic composites with responsive information display based on optical path control. Chem. Eng. J. **466**, 143286 (2023). 10.1016/j.cej.2023.143286

[27] J. Wang, H. Yu, J. Zheng, Y. Zhang, H. Guo et al., Nanograting-based dynamic structural colors using heterogeneous materials. Nano-Micro Lett. **17**(1), 59 (2024). 10.1007/s40820-024-01554-7 10.1007/s40820-024-01554-7 PMC11554963 39527350

[28] Y. Wu, Y. Wang, S. Zhang, S. Wu, Artificial chameleon skin with super-sensitive thermal and mechanochromic response. ACS Nano **15**(10), 15720–15729 (2021). 10.1021/acsnano.1c05612 34517702 10.1021/acsnano.1c05612

[29] S. Yu, D. Ma, C. Qi, D. Yang, S. Huang, All-in-one photonic crystals with multi-stimuli-chromic, color-recordable, self-healable, and adhesive functions. Adv. Funct. Mater. **34**(52), 2411670 (2024). 10.1002/adfm.202411670

[30] Y. Sun, X. Le, H. Shang, Y. Shen, Y. Wu et al., Dual-mode hydrogels with structural and fluorescent colors toward multistage secure information encryption. Adv. Mater. **36**(28), 2401589 (2024). 10.1002/adma.202401589 10.1002/adma.202401589 38744437

[31] Y. Shen, H. Shang, X. Le, Y. Wu, Y. Sun et al., Spatiotemporal regulation enabling photo-dimerizable gel networks toward multi-channel information encryption. Adv. Funct. Mater. **36**(2), e13532 (2026). 10.1002/adfm.202513532

[32] B. Liu, Z. Chen, J. Zhao, X. Gao, Y. Luo, Digitally programmable microphase separation in polymer network generates microstructure pattern. ACS Nano **18**(50), 34353–34362 (2024). 10.1021/acsnano.4c13111 39628292 10.1021/acsnano.4c13111

[33] Y. Huang, X. Ming, Z. Tang, G. Chen, X. Duan et al., Ultra-strong ionogel adhesives *via in situ* microphase separation. Adv. Funct. Mater. **35**(11), 2417011 (2025). 10.1002/adfm.202417011

[34] G.H. Lee, T.M. Choi, B. Kim, S.H. Han, J.M. Lee et al., Chameleon-inspired mechanochromic photonic films composed of non-close-packed colloidal arrays. ACS Nano **11**(11), 11350–11357 (2017). 10.1021/acsnano.7b05885 29095594 10.1021/acsnano.7b05885

[35] M. Kong, X. Guo, S. Zhang, Y. Zhang, B. Tang, Thermally-triggered structural color printing with excellent environmental tolerance. Adv. Funct. Mater. **35**(45), 2505714 (2025). 10.1002/adfm.202505714

[36] X. Zhang, T. Yin, J. Ge, Thermochromic photonic crystal paper with integrated multilayer structure and fast thermal response: a waterproof and mechanically stable material for structural-colored thermal printing. Adv. Mater. **36**, 2309344 (2024). 10.1002/adma.202309344 10.1002/adma.202309344 37906731

[37] Z. Zhao, H. Wang, L. Shang, Y. Yu, F. Fu et al., Bioinspired heterogeneous structural color stripes from capillaries. Adv. Mater. **29**(46), 1704569 (2017). 10.1002/adma.201704569 10.1002/adma.201704569 29044776

[38] D. Zhong, Z. Wang, J. Xu, J. Liu, R. Xiao et al., A strategy for tough and fatigue-resistant hydrogels *via* loose cross-linking and dense dehydration-induced entanglements. Nat. Commun. **15**, 5896 (2024). 10.1038/s41467-024-50364-3 39003311 10.1038/s41467-024-50364-3 PMC11246433

[39] D. Li, W. Zhan, W. Zuo, L. Li, J. Zhang et al., Elastic, tough and switchable swelling hydrogels with high entanglements and low crosslinks for water remediation. Chem. Eng. J. **450**, 138417 (2022). 10.1016/j.cej.2022.138417

[40] G. Yin, J. Wu, C. Qi, X. Zhou, Z.-Z. Yu et al., Pickering emulsion-driven MXene/silk fibroin hydrogels with programmable functional networks for EMI shielding and solar evaporation. Nano-Micro Lett. **17**(1), 312 (2025). 10.1007/s40820-025-01818-w 10.1007/s40820-025-01818-w PMC12185816 40551046

[41] Z. Zhang, Z. Chen, Y. Wang, Y. Zhao, L. Shang, Cholesteric cellulose liquid crystals with multifunctional structural colors. Adv. Funct. Mater. **32**(12), 2107242 (2022). 10.1002/adfm.202107242

[42] X. Wen, Y. Yue, C. Wang, J. Zhang, Y. Xie et al., Bio-inspired cellulose composites with multicolor separation *via* electro-thermal and magneto-thermal techniques for multifunctional applications. Adv. Funct. Mater. **34**(48), 2408792 (2024). 10.1002/adfm.202408792

[43] Y. Wang, L. Sun, G. Chen, H. Chen, Y. Zhao, Structural color ionic hydrogel patches for wound management. ACS Nano **17**(2), 1437–1447 (2023). 10.1021/acsnano.2c10142 10.1021/acsnano.2c10142 36512760

[44] H. Fukumoto, K. Ishihara, S.-I. Yusa, Thermo-responsive behavior of mixed aqueous solution of hydrophilic polymer with pendant phosphorylcholine group and poly(acrylic acid). Polymers **13**, 148 (2021). 10.3390/polym13010148 33401453 10.3390/polym13010148 PMC7794920

[45] J. Yuan, C. Li, S. Wang, H. Zhang, Z. Wang et al., Methods and characteristics of drug extraction from ion-exchange-resin-mediated preparations: influences, thermodynamics, and kinetics. Polymers **15**, 1191 (2023). 10.3390/polym15051191 36904432 10.3390/polym15051191 PMC10007538

[46] Y. Liu, Y. Lei, L. Hua, J. Lu, K. Wang et al., Biomimetic self-deformation of polymer interpenetrating network with stretch-induced anisotropicity. Chem. Mater. **33**(21), 8351–8359 (2021). 10.1021/acs.chemmater.1c02639

[47] L. Hua, M. Xie, Y. Jian, B. Wu, C. Chen et al., Multiple-responsive and amphibious hydrogel actuator based on asymmetric UCST-type volume phase transition. ACS Appl. Mater. Interfaces **11**(46), 43641–43648 (2019). 10.1021/acsami.9b17159 31663325 10.1021/acsami.9b17159

[48] F. Meng, B. Ju, Z. Wang, R. Han, Y. Zhang et al., Bioinspired polypeptide photonic films with tunable structural color. J. Am. Chem. Soc. **144**(17), 7610–7615 (2022). 10.1021/jacs.2c02894 35446030 10.1021/jacs.2c02894

[49] K.Y. Chung, B. Xu, D. Tan, Q. Yang, Z. Li et al., Naturally crosslinked biocompatible carbonaceous liquid metal aqueous ink printing wearable electronics for multi-sensing and energy harvesting. Nano-Micro Lett. **16**(1), 149 (2024). 10.1007/s40820-024-01362-z 10.1007/s40820-024-01362-z PMC10928061 38466478

[50] J. Zhou, X. Allonas, A. Ibrahim, X. Liu, Progress in the development of polymeric and multifunctional photoinitiators. Prog. Polym. Sci. **99**, 101165 (2019). 10.1016/j.progpolymsci.2019.101165

[51] S. Ma, H. Qie, X. Yang, X. Yang, Q. Zhang et al., Damping supramolecular elastomer for steady hypothermic sensing. Adv. Funct. Mater. **35**(29), 2424996 (2025). 10.1002/adfm.202424996

[52] Y. Qi, L. Song, C. Zhou, S. Zhang, Hydration activates dual-confined shape-memory effects of cold-reprogrammable photonic crystals. Adv. Mater. **35**(16), 2210753 (2023). 10.1002/adma.202210753 10.1002/adma.202210753 36658743

[53] P.L. Ritger, N.A. Peppas, A simple equation for description of solute release II. Fickian and anomalous release from swellable devices. J. Control. Release **5**(1), 37–42 (1987). 10.1016/0168-3659(87)90035-6

