# Supplementary material for: Information Security with Smart Hydrogels: Photo-Patterning and Multi-Stimuli Responsive Structural Color
Source: Nanomicro Lett. 2026 Mar 31;18:311. doi: 10.1007/s40820-026-02130-x (PMC13038763; doi:10.1007/s40820-026-02130-x)
Supplement: Supplementary file 1 — Supplementary file1 (DOCX 11103 KB) [file 40820_2026_2130_MOESM1_ESM.docx]

Supporting Information for

**Information Security with Smart Hydrogels: Photo-patterning and Multi-stimuli Responsive Structural Color**

Xiaoyu Guo^1^, Ying Li^1^, Farzana Hanif^1^, Linhai Zhu^1^, Miao Kong^1^, Shufen Zhang^1^, Yuang Zhang^1^, and Bingtao Tang^1,^ *

^1^State Key Laboratory of Fine Chemicals, Frontiers Science Center for Smart Materials Oriented Chemical Engineering, Dalian University of Technology, Dalian 116024, P. R. China

*Corresponding author. [tangbt@dlut.edu.cn](mailto:tangbt@dlut.edu.cn) (Bingtao Tang)

## S1 Experimental Sections

### S1.1 Materials

Tetraethyl orthosilicate (AR, ≥99%) was purchased from Xilong Chemical Reagent Factory. Ammonia solution (25 wt%) and hydrofluoric acid (4 wt%)were bought from Tianjin Damao Chemical Reagent Factory. Absolute ethanol was obtained from Sinopharm Chemical Reagent Co., Ltd. Acrylic acid (AA) (AR, ≥99%) and acrylic amide (AM) (AR, ≥97%), 4-acryloyloxy benzophenone (ABP), N, N-methylene acrylamide (MBAA) (AR, ≥97%) and azo diisobutyronitrile (AIBN) (AR, ≥90%) were bought from Aladdin. All reagents and solvents were used as obtained.

### S1.2 Characterization

SEM images of SiO_2_ nanospheres and photonic films were observed by a scanning electron microscope (Nova Nano SEM 450), and the elemental mapping was performed by its supporting energy-dispersive X-ray spectrometer. The morphology of the films under different conditions was characterized by atomic force microscopy (AFM, XE-70, PARK Instruments, Korea). The normal-incidence reflection spectra were measured by an ocean spectrometer QE Pro 2000. Transmission spectroscopy was tested using Series UV-Vis-NIR Spectrophotometer (Agilent) with a sample thickness of 0.3mm. Photos of all samples were taken with an honor magic7 smartphone. The monomer precursor was cured into by portable UV lamp (WFH-204B, Shanghai Jingke Industrial Co., Ltd). The infrared reflection spectra were recorded by nicolet IS50. XPS spectra were characterized by an ESCA-LAB250 multifunction surface analysis system. The thermal stability of hydrophobic photonic film was measured at 10 °C min^-1^ under N_2_ atmosphere using a TGA Q500 instrument. The tensile strength test was conducted with the PT-305 Computer universe testing machine from Dongguan Precise-test Equipment Co., Ltd. The glass transition temperature (Tg) was obtained by testing sample strips with a length and width of 3×0.5 cm^2^ and a thickness of 0.3 mm using Dynamic Mechanical Analyzers (Q800).

Bragg diffraction Eqs. [S1-S3]:

$\lambda=1.633D{({n_{eff}}^{2}-{sin}^{2}\theta)}^{1/2}$ [S1]

where D is the center-to-center distance of the nanoholes, n_eff_ is the average refractive index of the photonic material, and θ is the angle of incidence. Therefore, adjusting the structural color by modulating the lattice spacing or refractive index is feasible.

Film swelling test: place the dry film with an initial mass of m_0_ in water. Take it out after 30 s of soaking, wipe off the surface moisture with a paper towel, quickly weigh its mass as m_t_, and continue to soak until the mass hardly changes and stabilizes at m_∞_. The test results are shown in Fig. S11.

F=$\frac{M_{t}-M_{0}}{M_{\infty}-M_{0}}$ [S2]

ln F=a ln t + ln k [S3]

Which F water retention rates, says 𝑀 up said balance the quality (soaking 24 h), k for swelling constant, according to a limit value, can assume that three theoretical model: 1) a < 0.5, Fickian type, can be interpreted as the free diffusion solvent molecules; 2) 0.5 < a < 1, non-Fickian type, the diffusion of the solvent and the diffusion of the molecular chain jointly control the adsorption of the solvent; 3) When a > 1, the diffusion system is controlled by the extension of the polymer chain [S4,S5].

## S2 Supplementary Figures and Tables

**Table S1.** The particle size, mono-dispersion and surface potential of SiO_2_ microspheres

| sample | Seed dosage  (mL) | diameter  (nm) | polydispersity index  (PDI) | Zeta potential (mV) |
| --- | --- | --- | --- | --- |
| 1 | 0.5 | 354 | 0.046 | -42.3 |
| 2 | 0.6 | 301 | 0.028 | -38.2 |
| 3 | 0.8 | 285 | 0.019 | -44.6 |
| 4 | 1.0 | 273 | 0.035 | -35.8 |
| 5 | 1.5 | 243 | 0.024 | -39.2 |
| 6 | 2.0 | 225 | 0.036 | -34.1 |


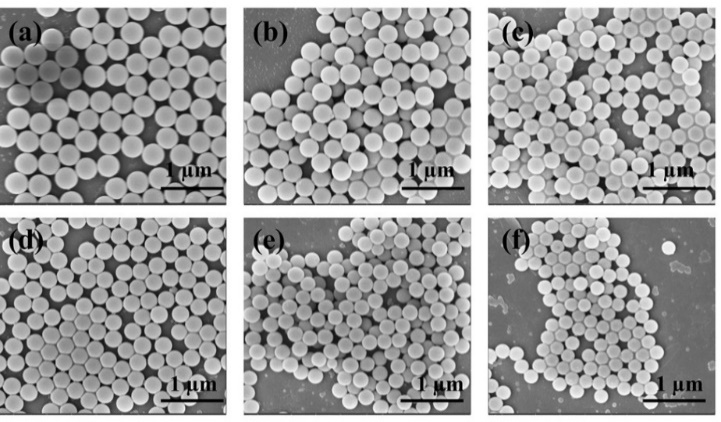


**Fig. S1** SEM image of SiO_2_ colloidal microspheres **a** 354 nm, **b** 301 nm, **c** 285 nm, **d** 273 nm, **e** 243nm, **f** 225 nm


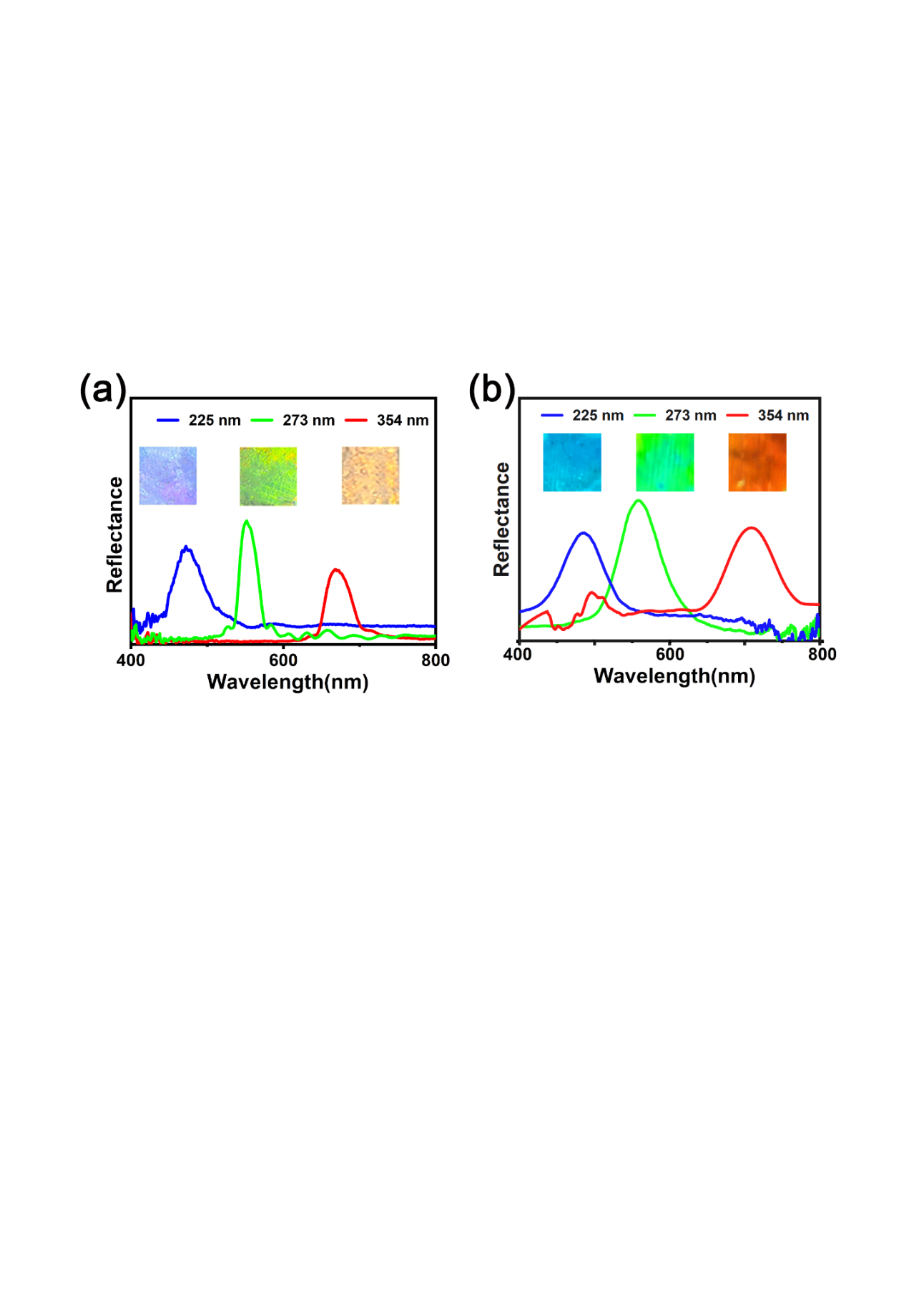


**Fig.** **S2** Optical photographs and reflection spectra of **a** opal template and **b** anti-opal hydrogel structural colors


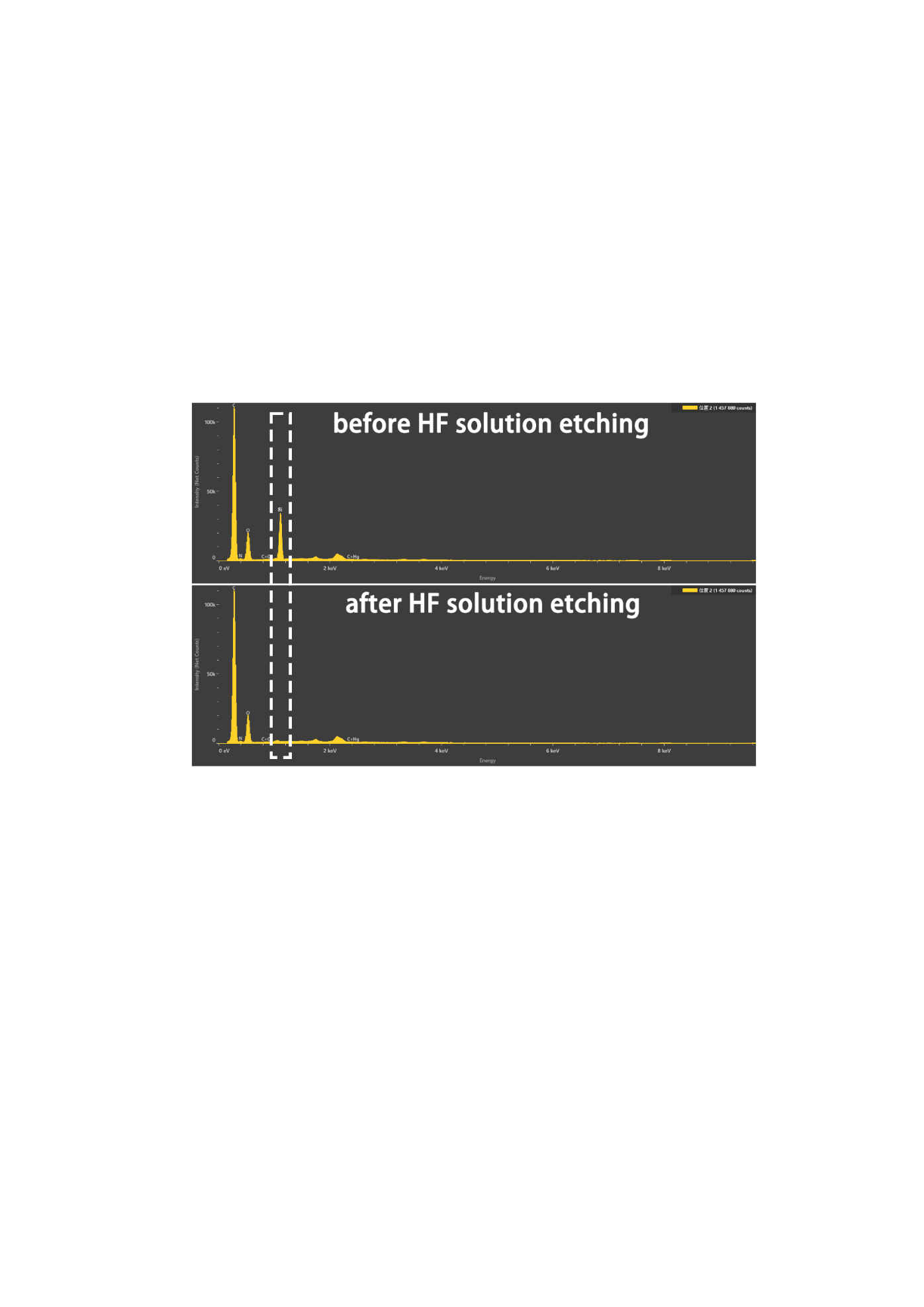


**Fig.** **S3** EDS spectra before and after etching


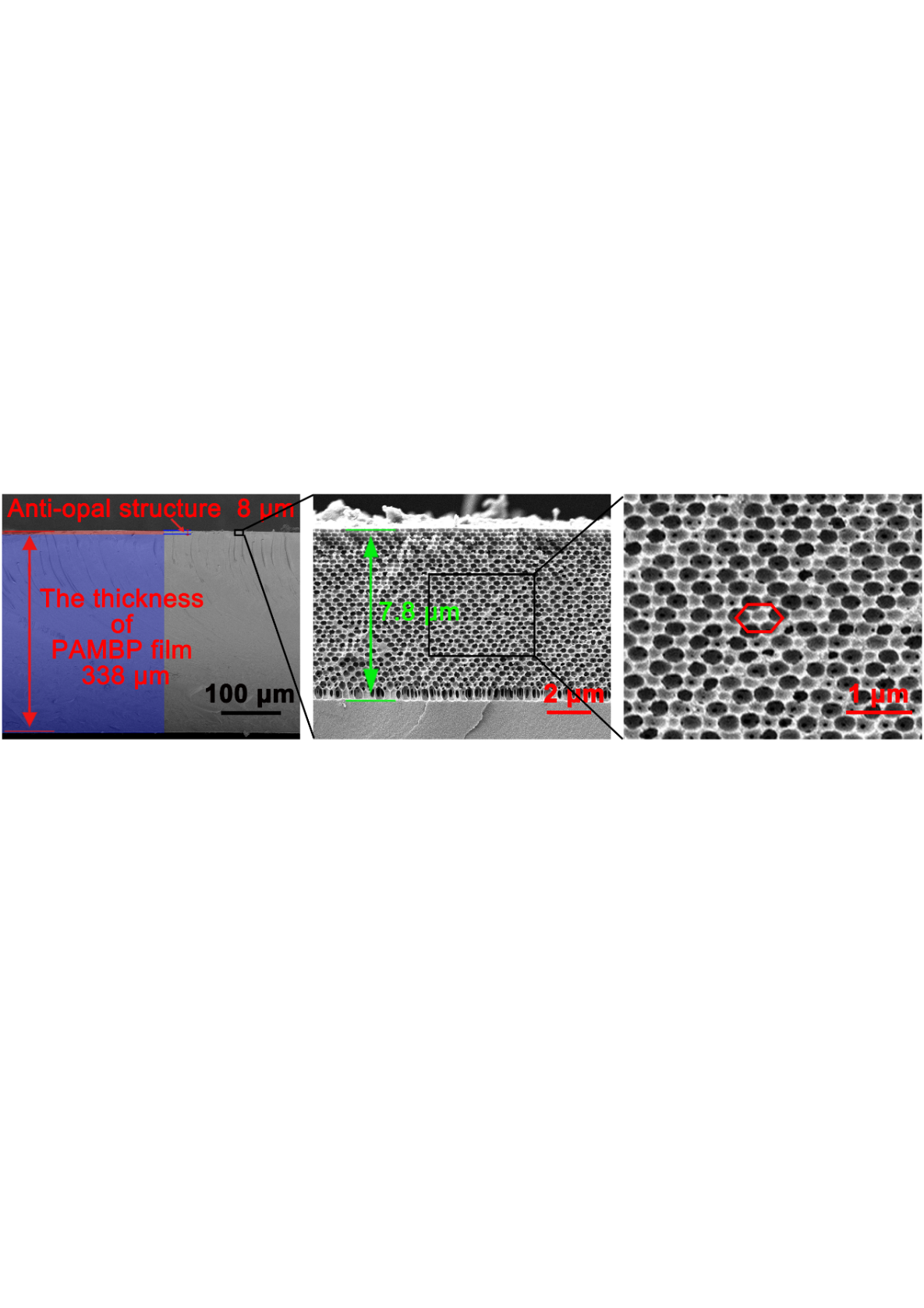


**Fig.** **S4** Cross-sectional SEM image of PAMBP anti-opal structure color film


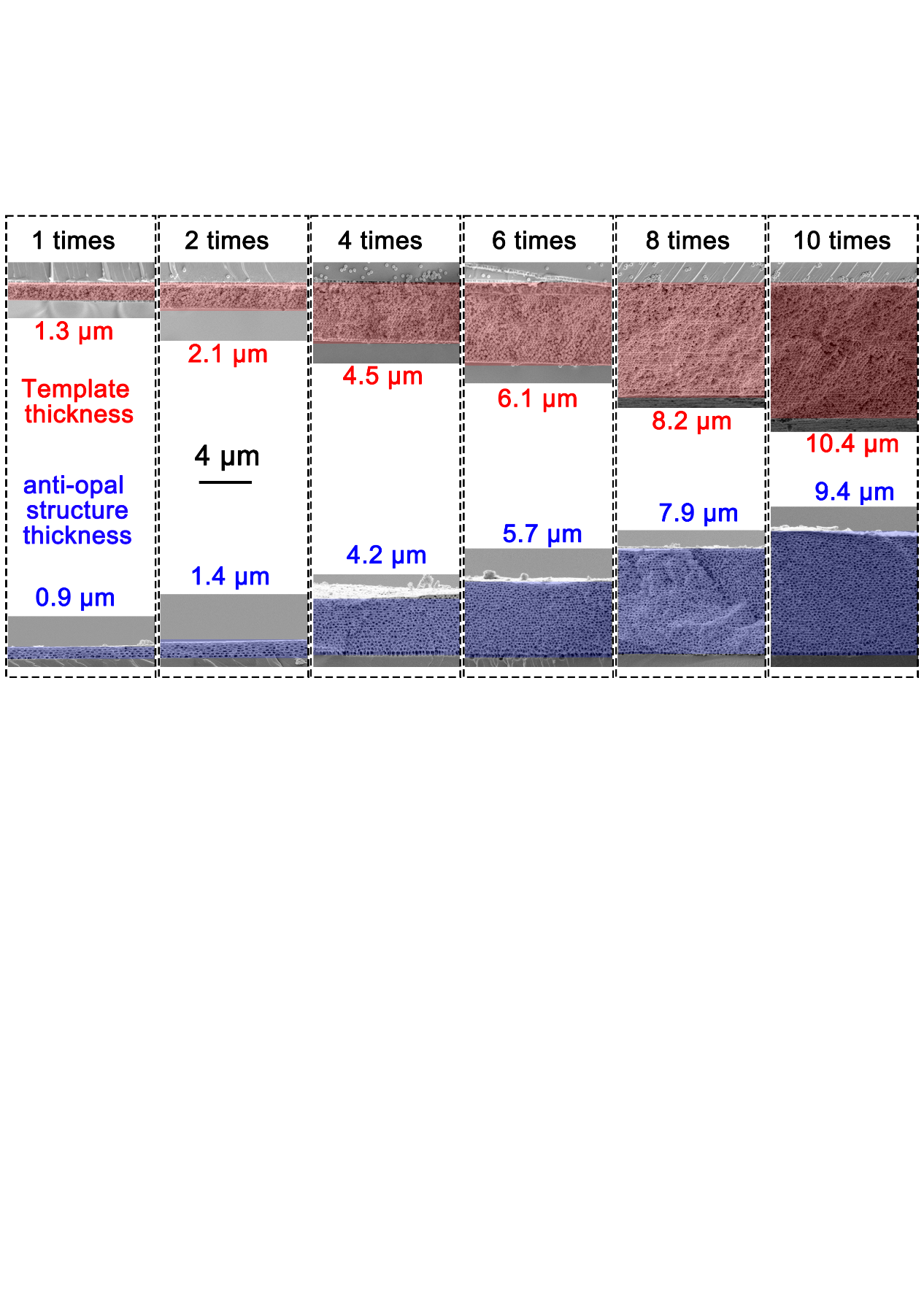


**Fig. S5** SEM images of the cross-sections of the templates and anti-opal structures obtained by varying the number of coating and pulling processes


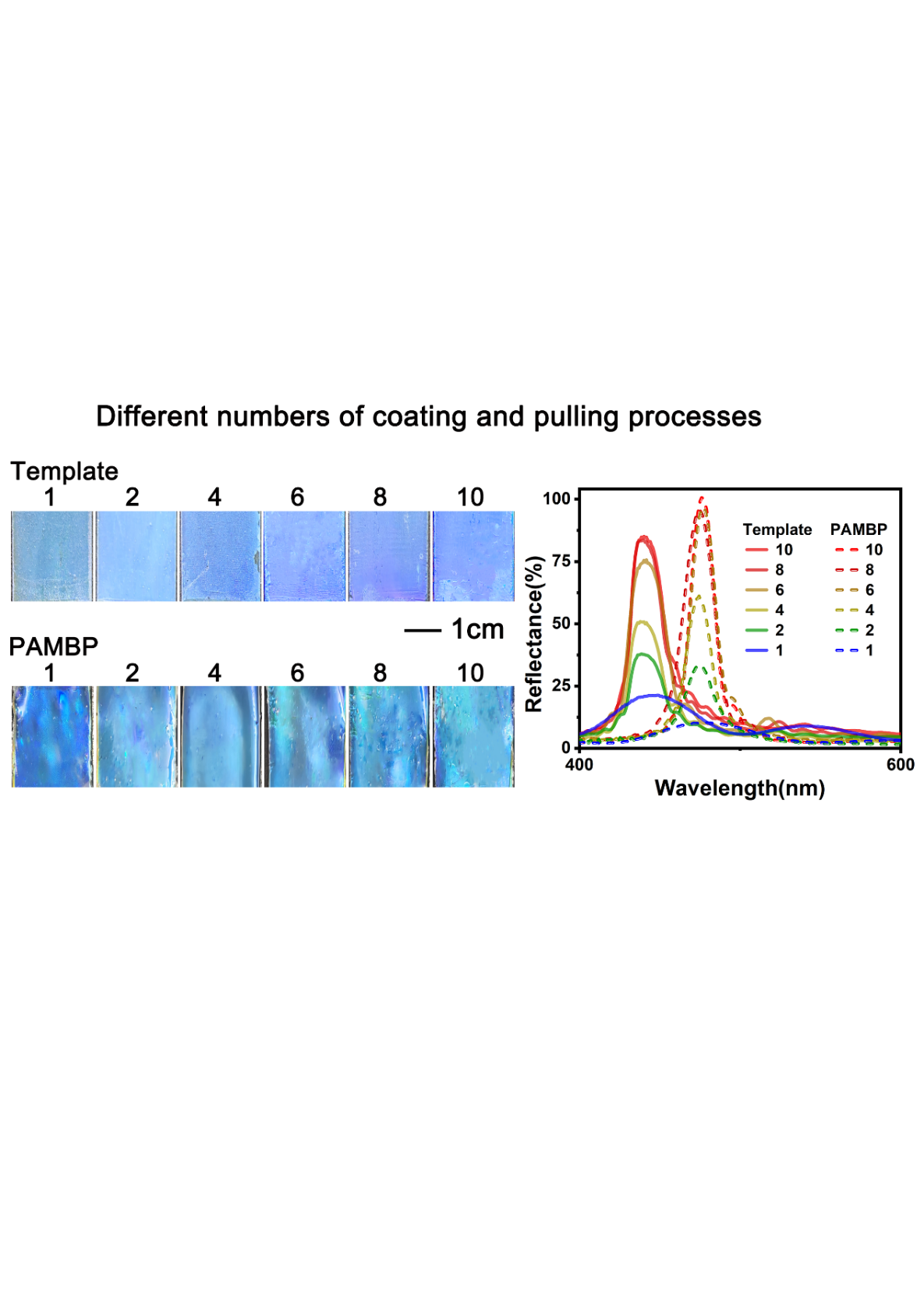


**Fig. S6** Optical photos and reflection spectra of the cross-sections of the templates and anti-opal structures obtained by varying the number of coating and pulling processes


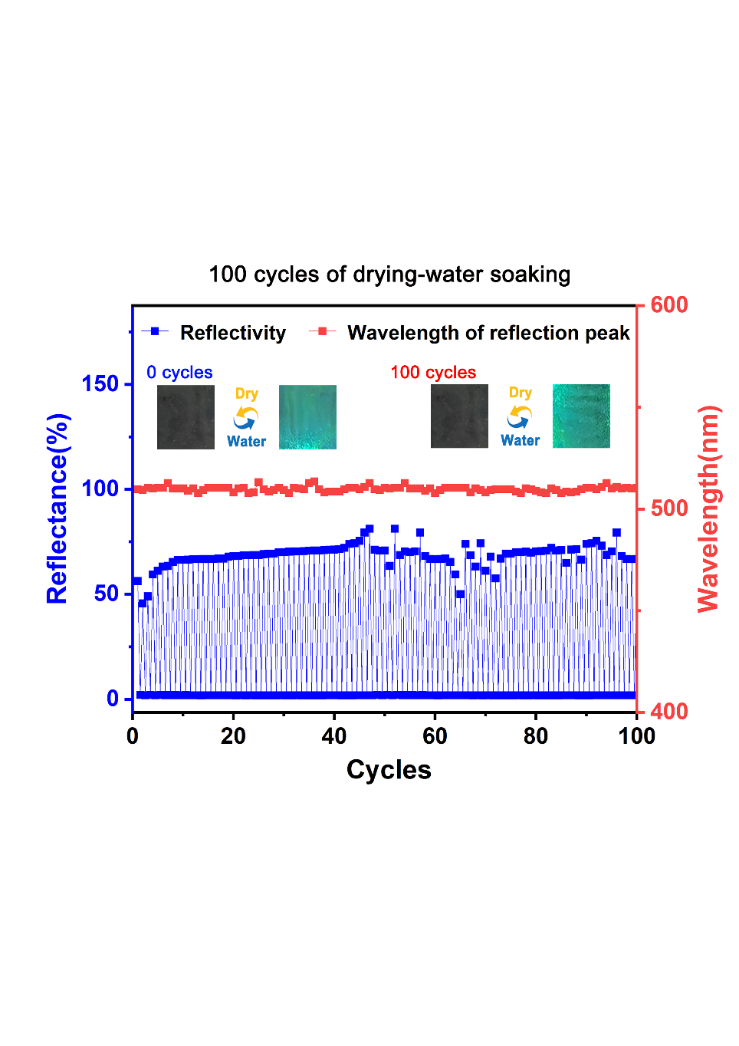


**Fig.** **S7** The reflectance and maximum reflection wavelength variation graph of PAMBP during 100 dry - water soaking cycles


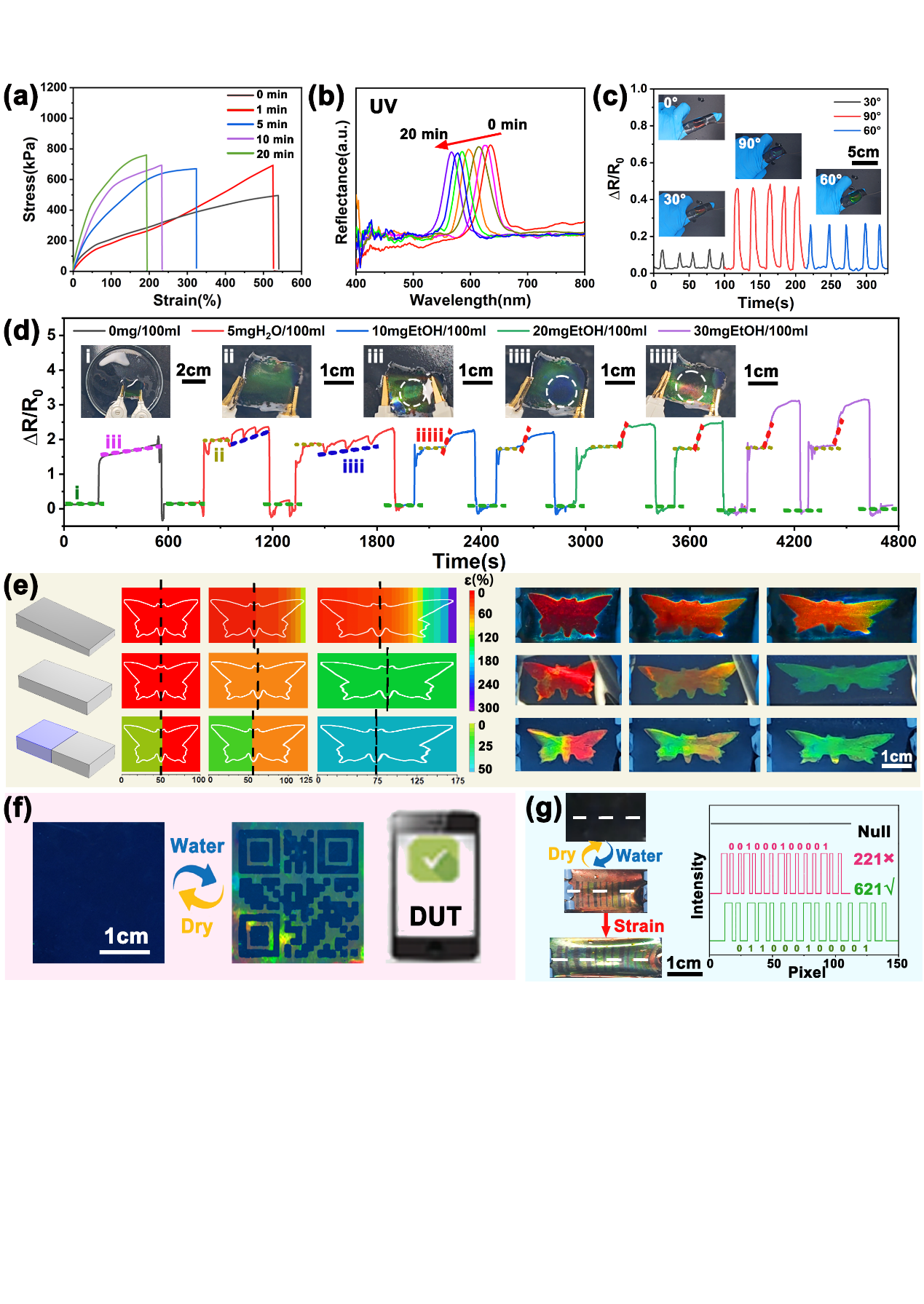


**Fig. S8** Optical photos of the water-induced color development of PAMBP films patterned with QR codes


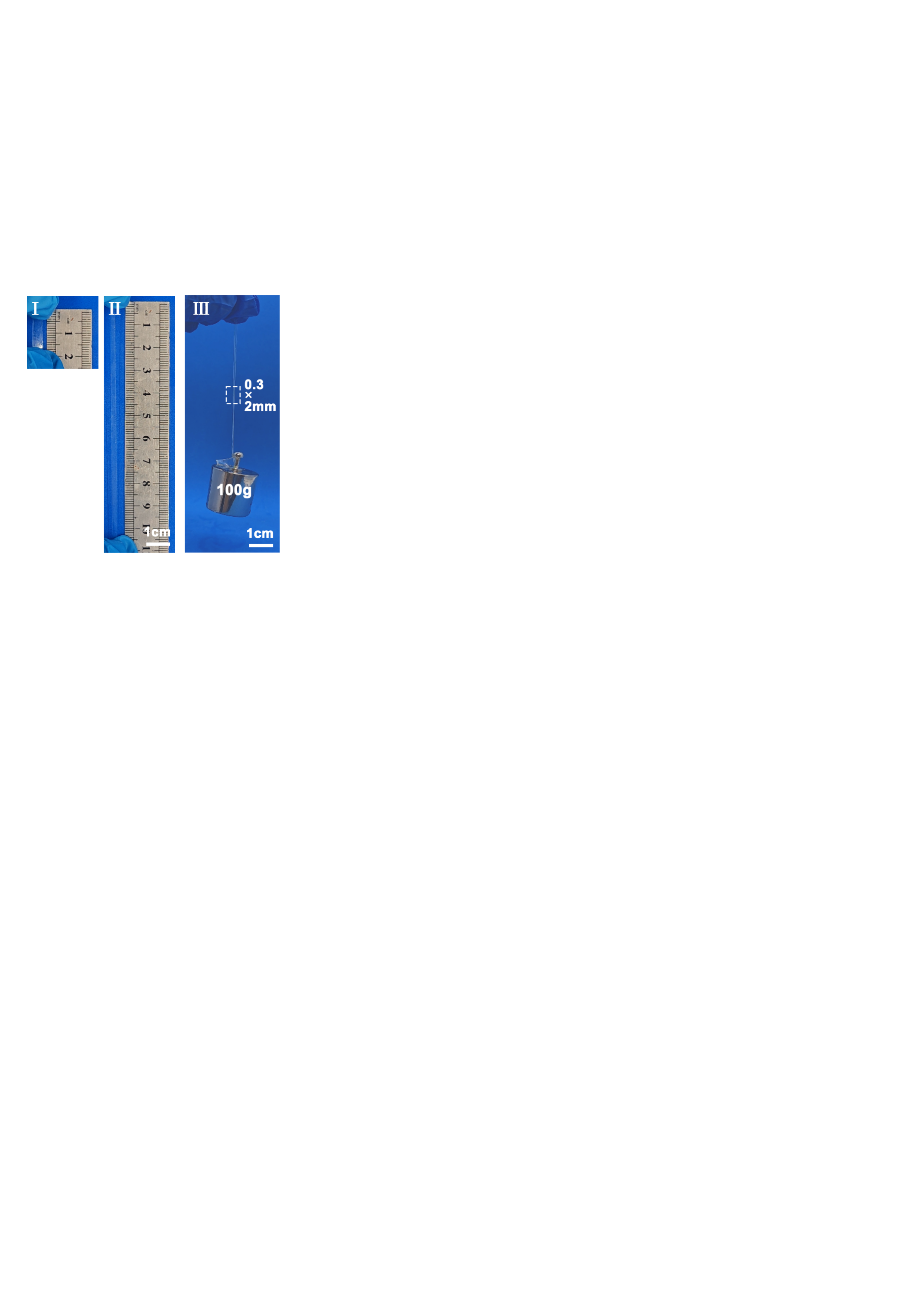


**Fig. S9** Optical photos of P621 tensile test and P621 lifting a 100g weight


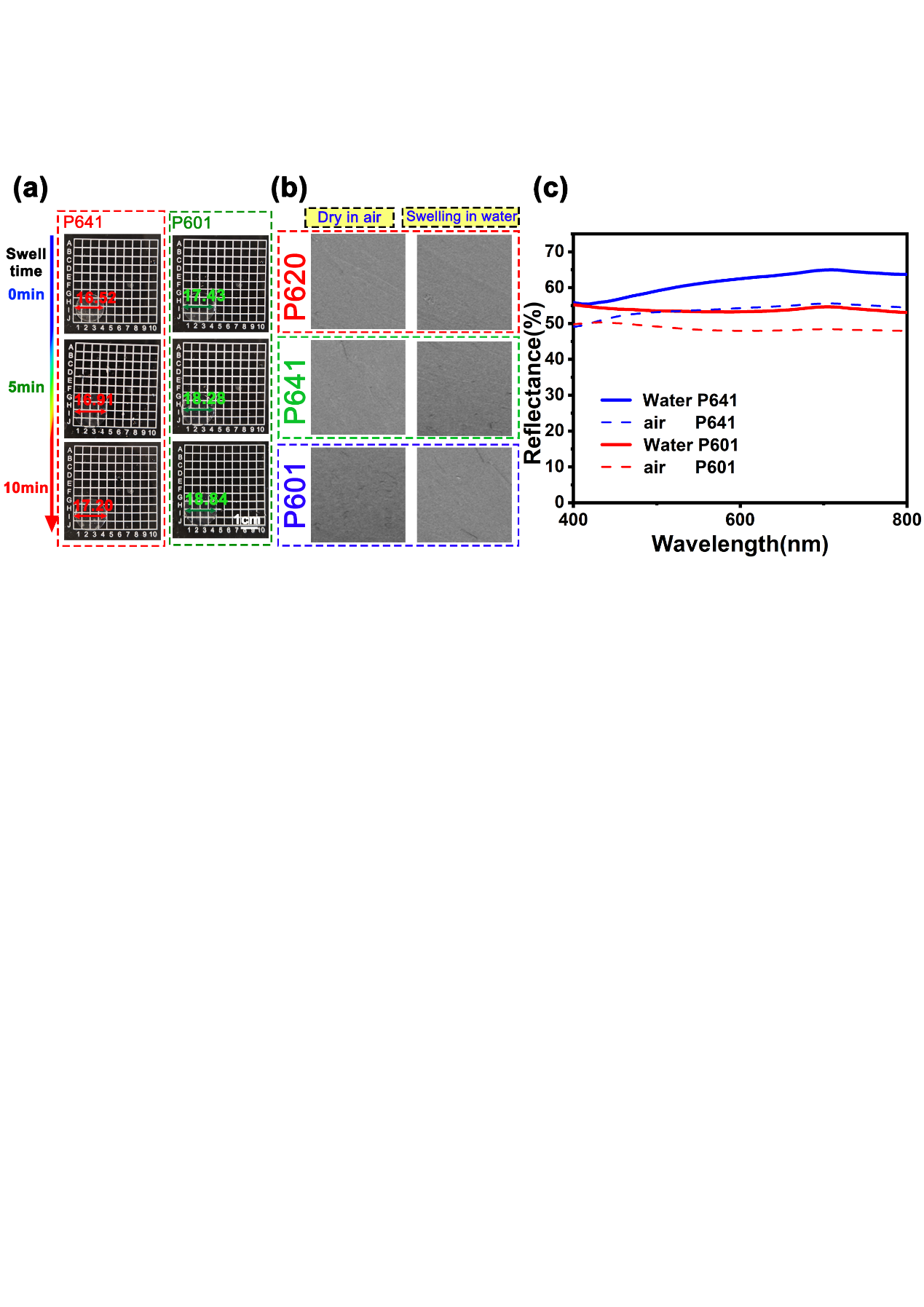


**Fig. S10** **a** Optical images of P641 and P601 swelling in deionized water at 0 min, 5 min, and 10 min respectively. **b** Cross-sectional SEM images of P620, P641 and P601 in a dry state and after being thoroughly soaked in deionized water and freeze-dried. **c** Transmission spectra of P641 and P601 under dry conditions and after being thoroughly soaked in deionized water


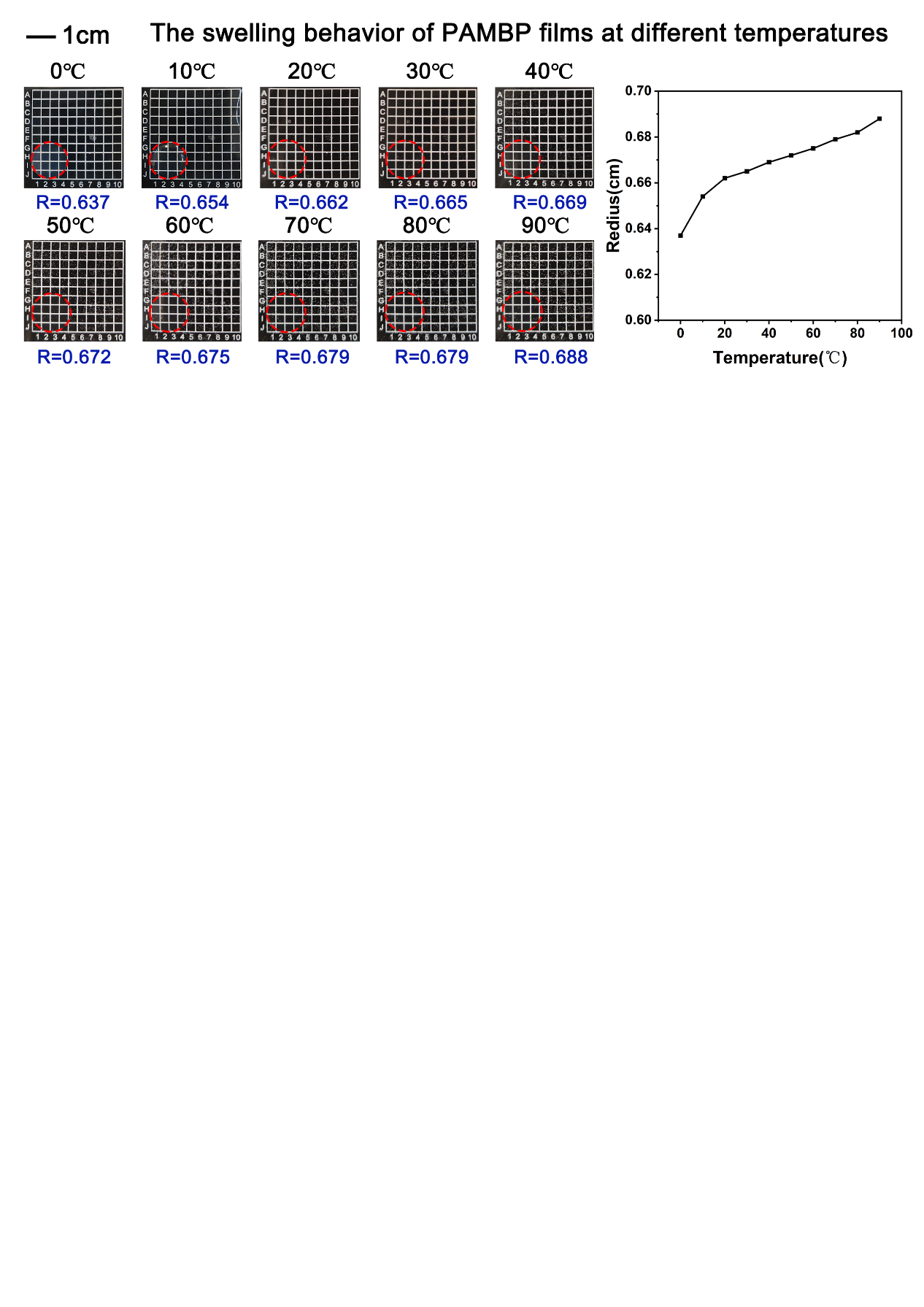


**Fig. S11** Optical photos of the circular PAMBP films at different temperatures during the swelling equilibrium process and the radius-temperature line graph


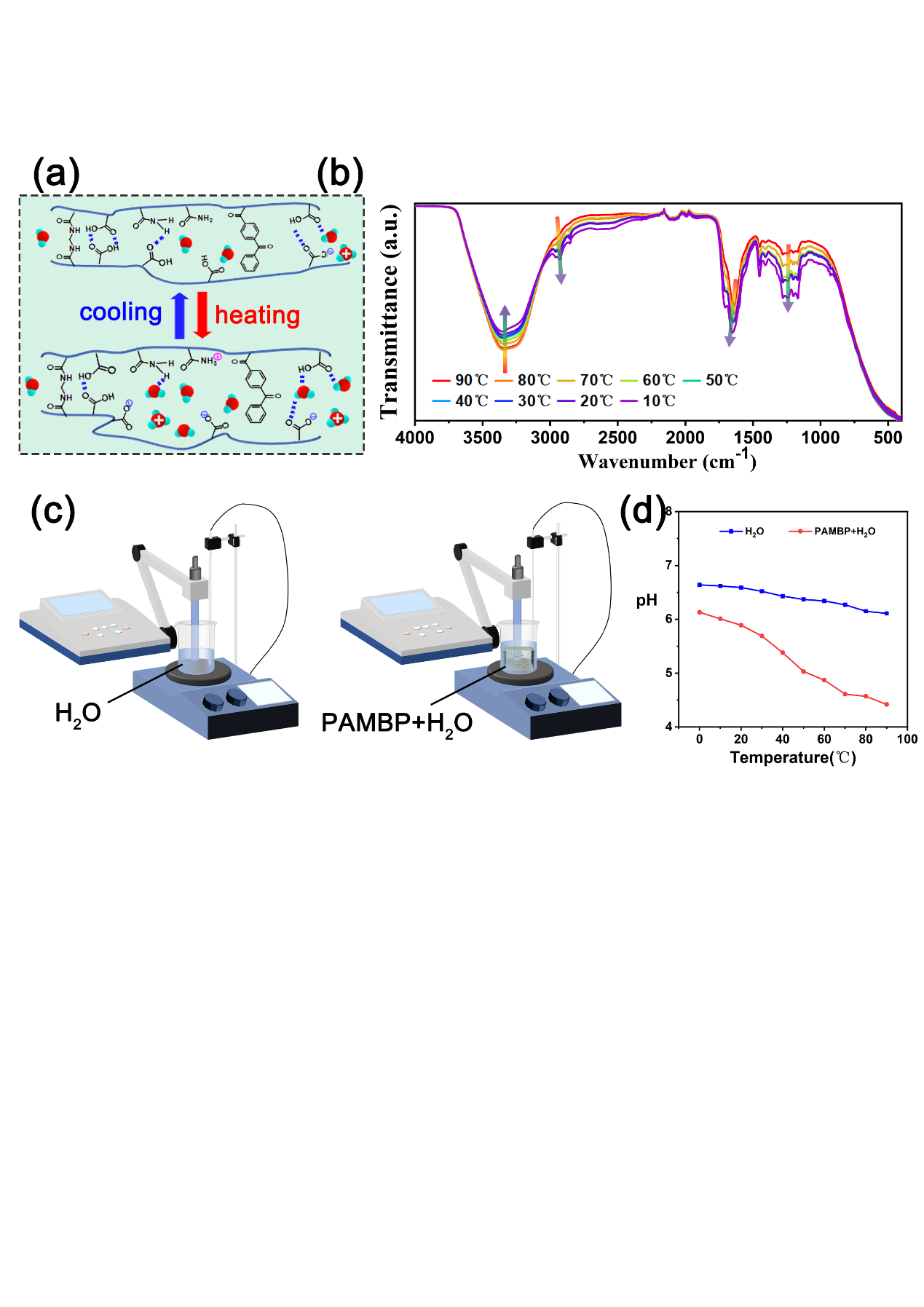


**Fig. S12** **a** Schematic diagram of possible changes in molecular chain structure as the degree of swelling increases with rising temperature. **b** The temperature-dependent infrared spectrum of PAMBP. **c** Schematic diagram of the pH testing method for the H_2_O and PAMBP + H_2_O mixture when the temperature changes. **d** The pH-temperature graph of H_2_O and PAMBP + H_2_O mixture


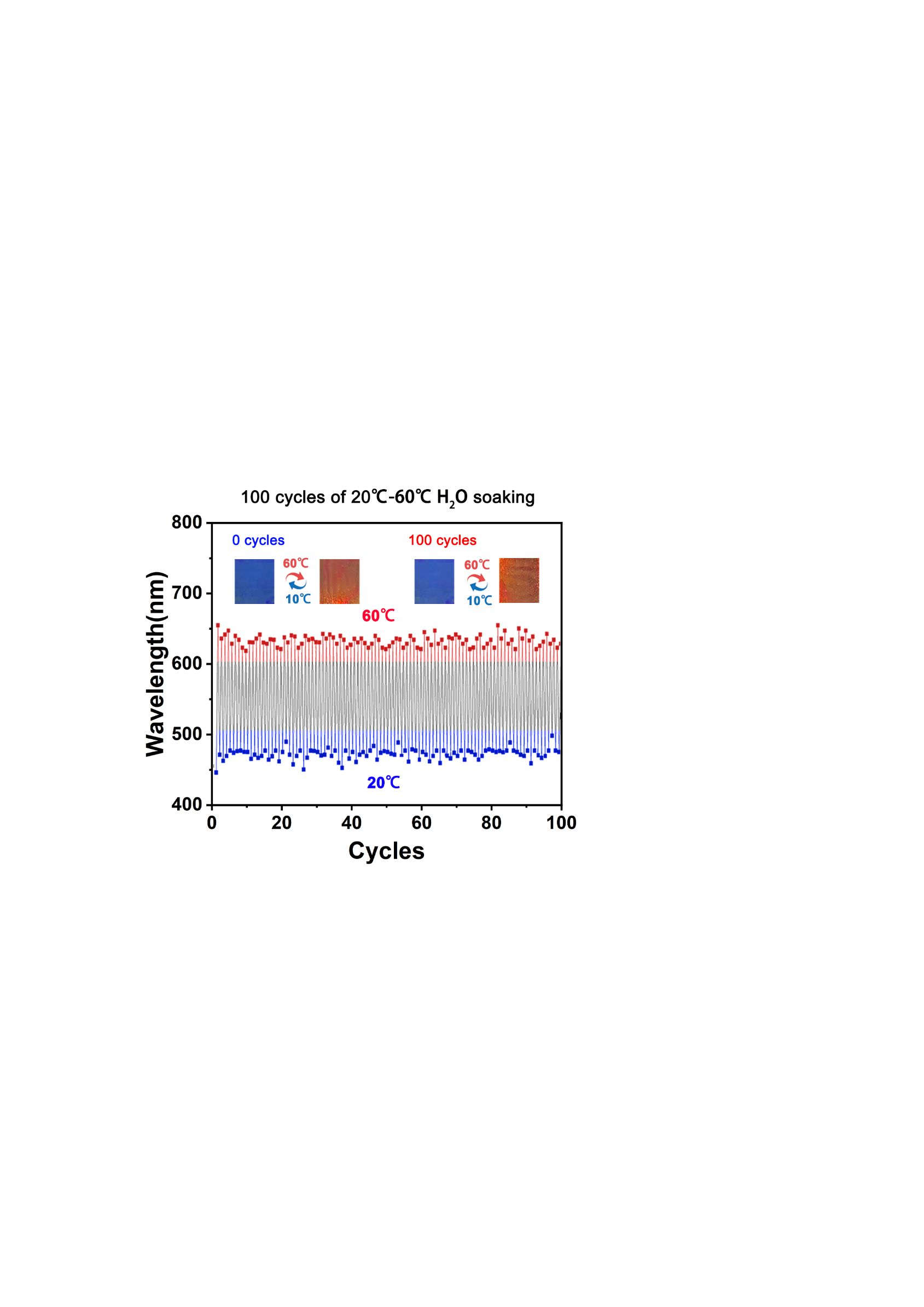


**Fig. S13** The wavelength variation diagram of the maximum reflectance of PAMBP during 100 cold and hot cycles


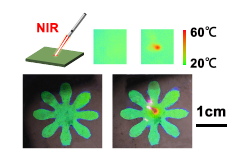


**Fig.** **S14** Schematic diagrams, optical photos and infrared thermal imaging photos of PAMBP's infrared light response.


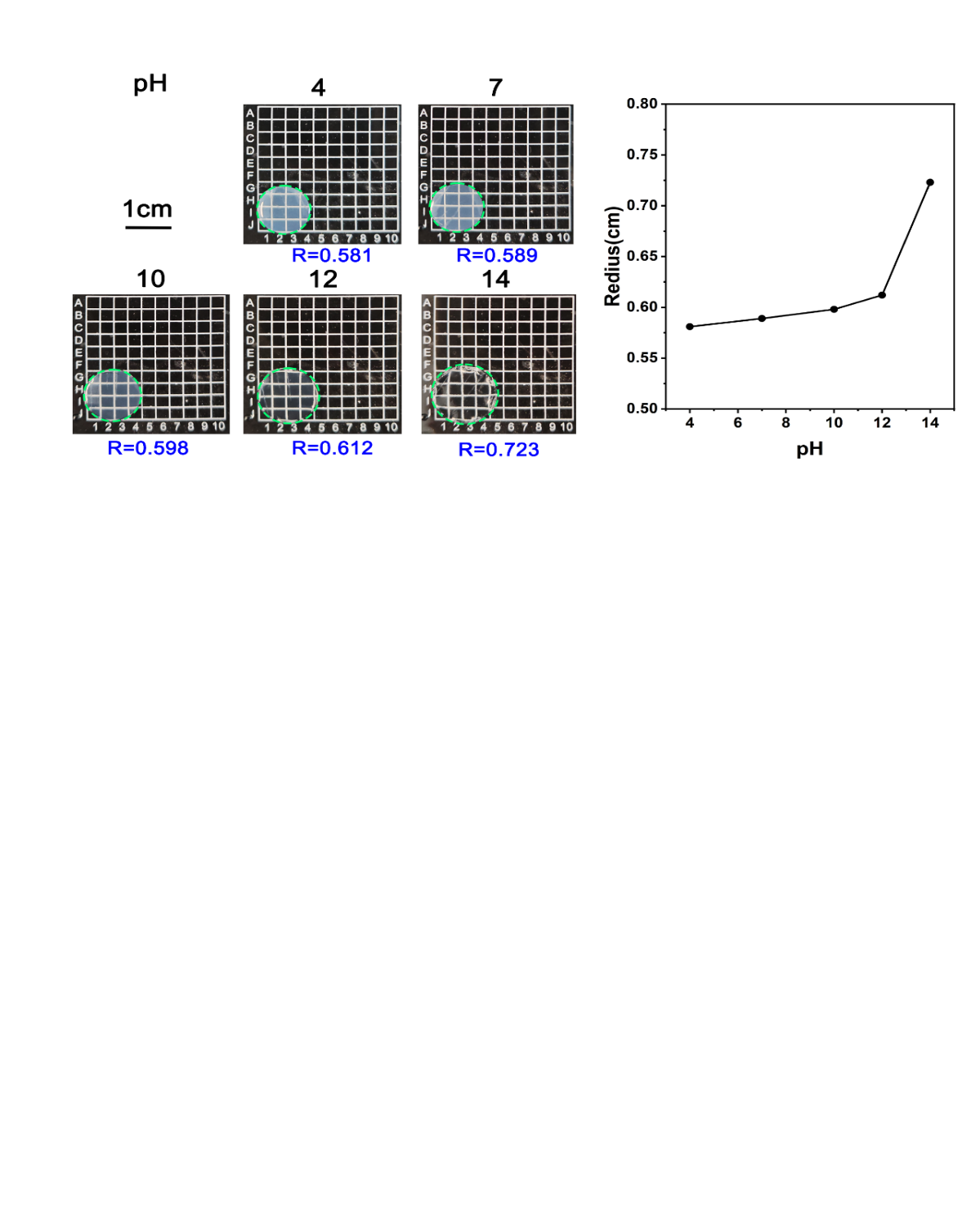


**Fig. S15** Optical photos of the circular PAMBP films at different pH during the swelling equilibrium process and the radius-temperature line graph


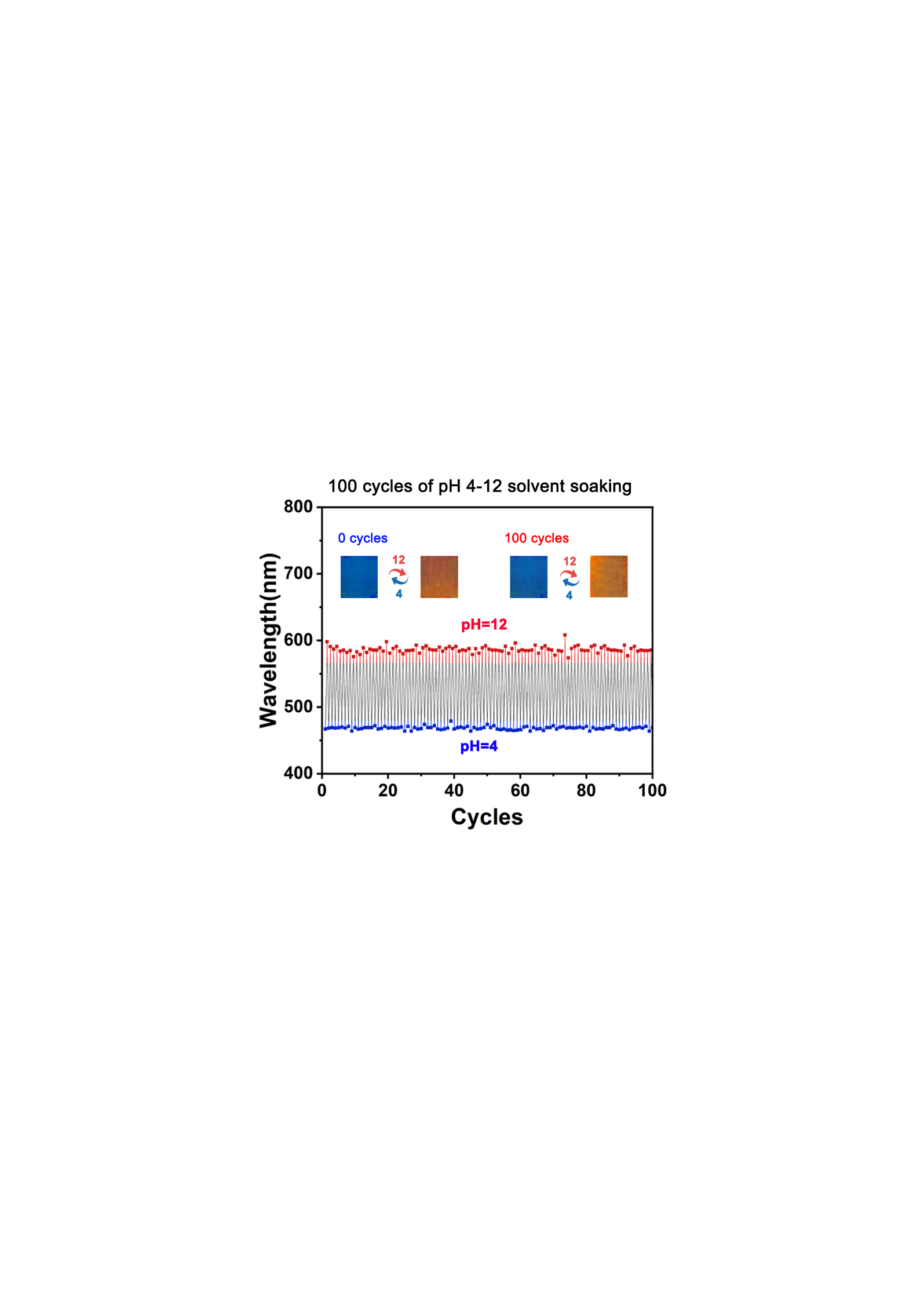


**Fig. S16** The wavelength variation diagram of the maximum reflectance of PAMBP during 100 solvent immersion cycles with pH values of 4 and 12


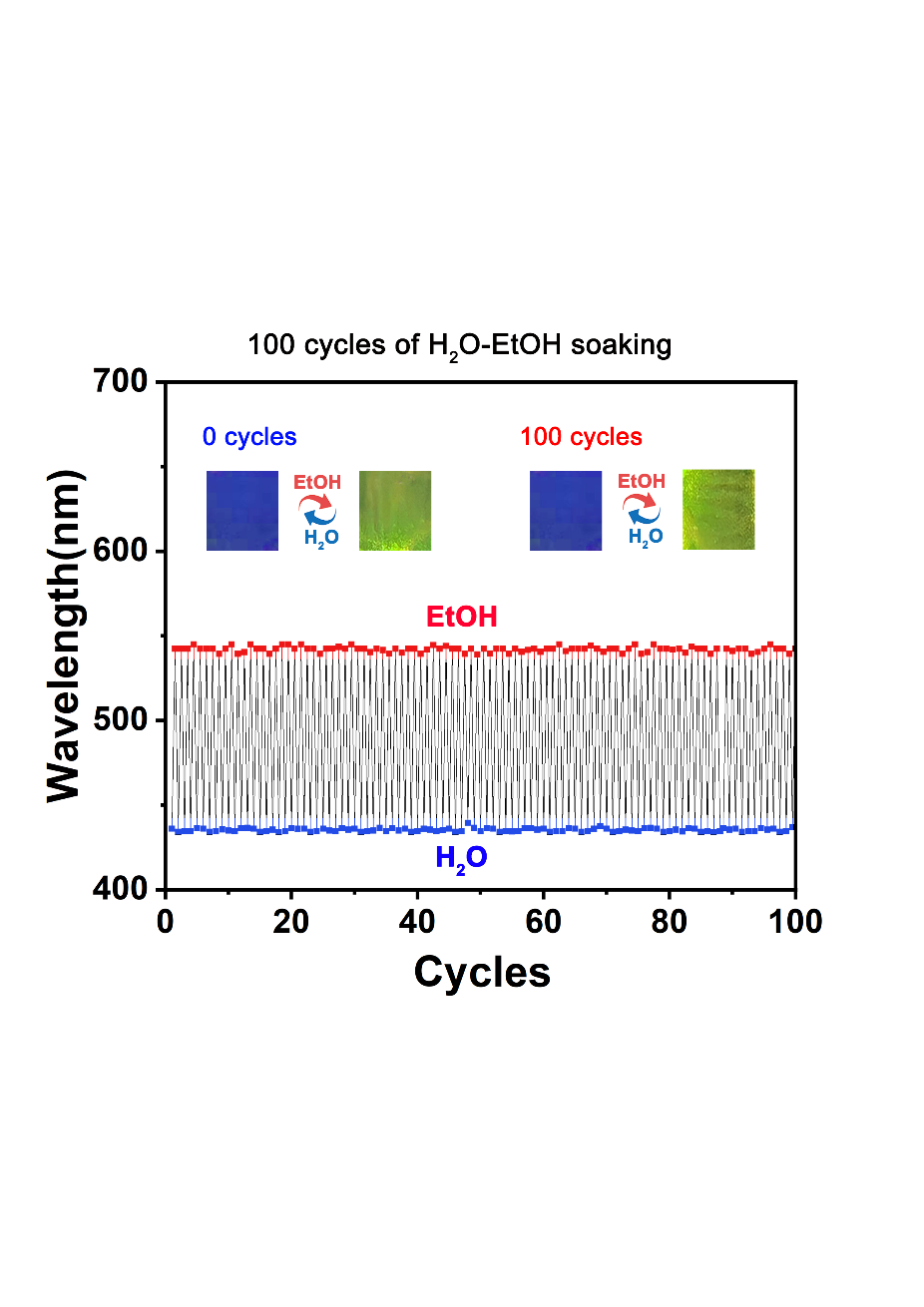


**Fig. S17** The wavelength variation diagram of the maximum reflectance of PAMBP during 100 cycles of water and ethanol soaking


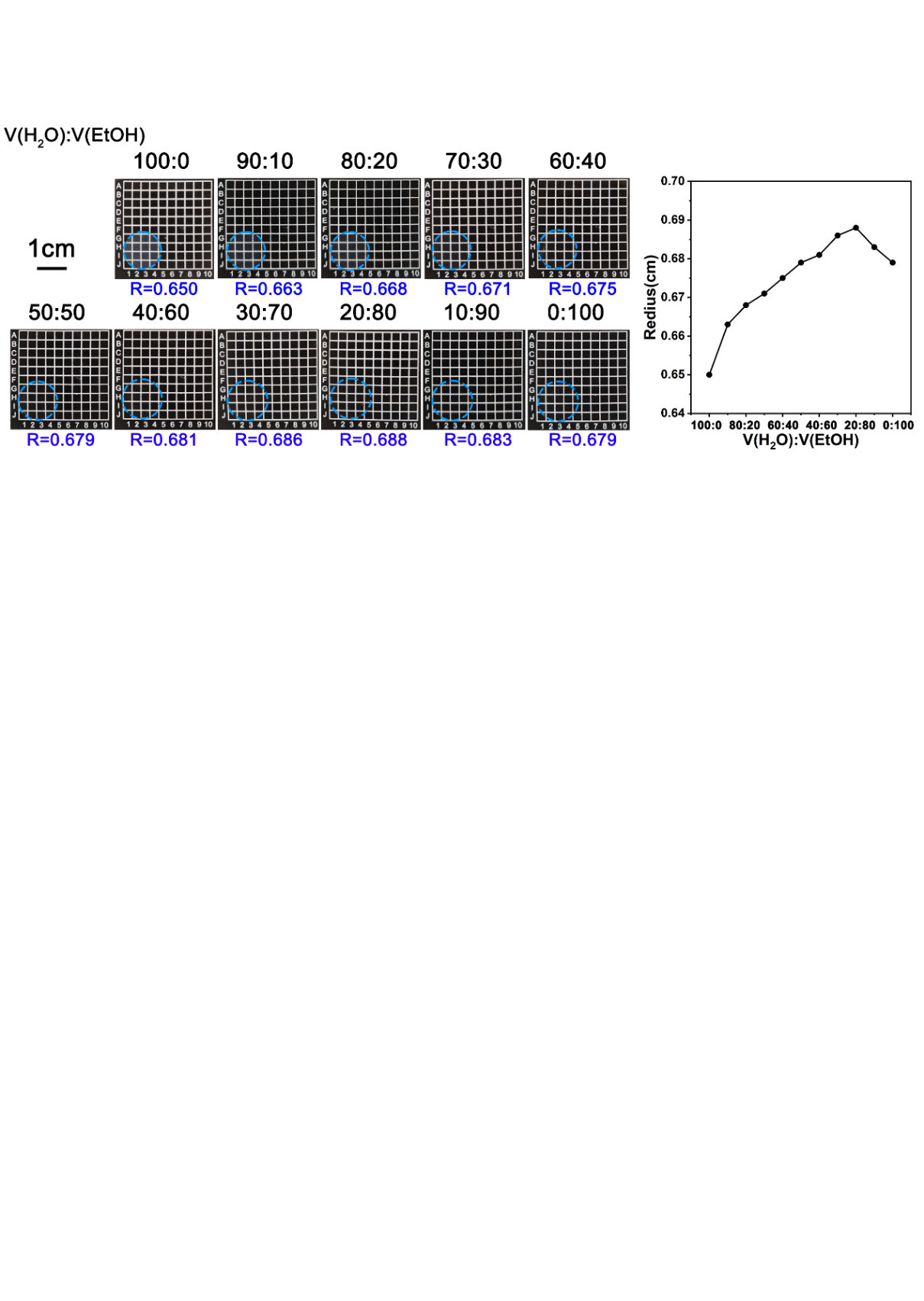


**Fig. S18** Optical photos of the circular PAMBP films at different alcohol-water concentration during the swelling equilibrium process and the radius-temperature line graph


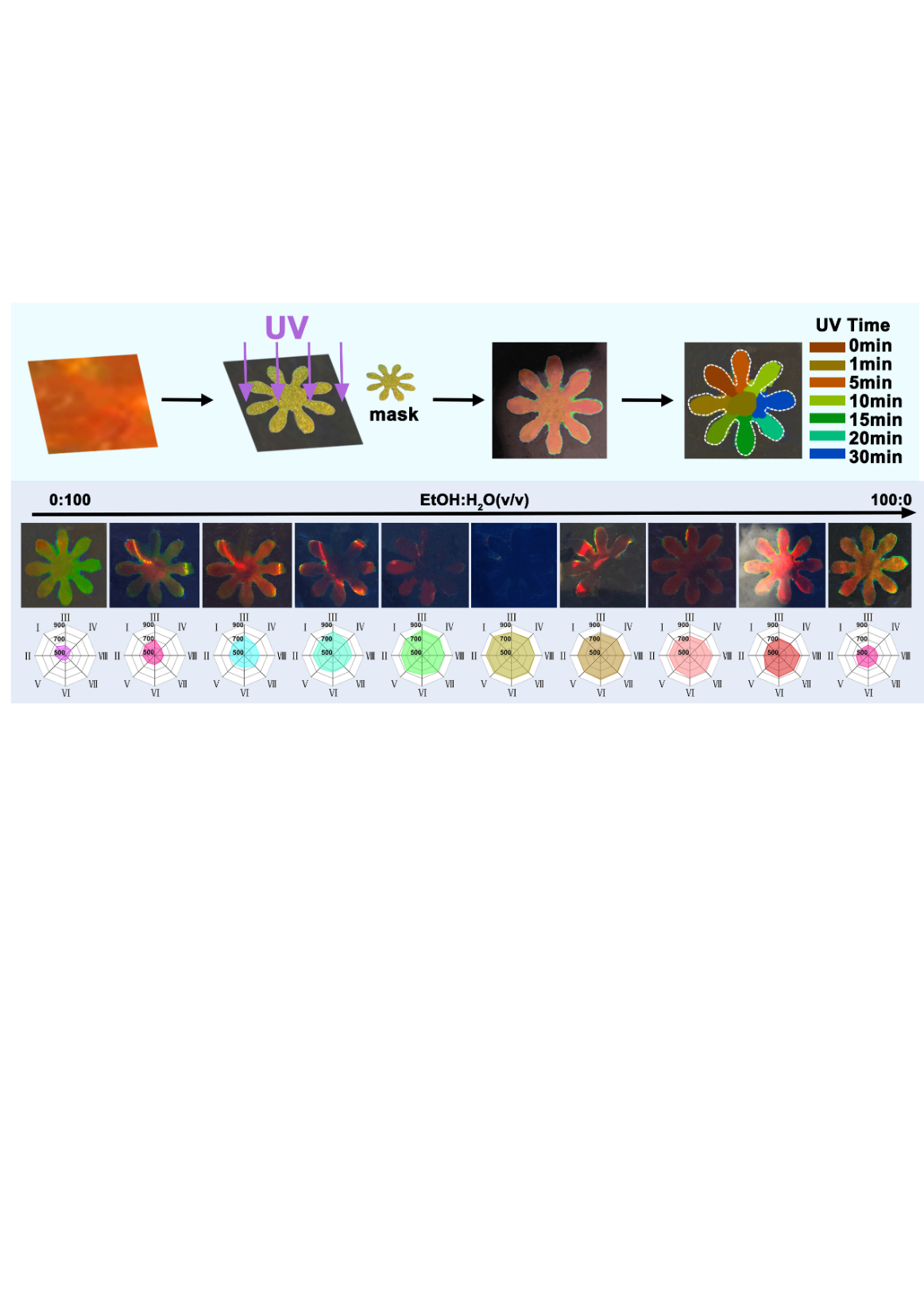


**Fig. S19** Schematic diagram of the preparation of ethanol concentration sensing array PAMBP and optical image of alcohol-water response


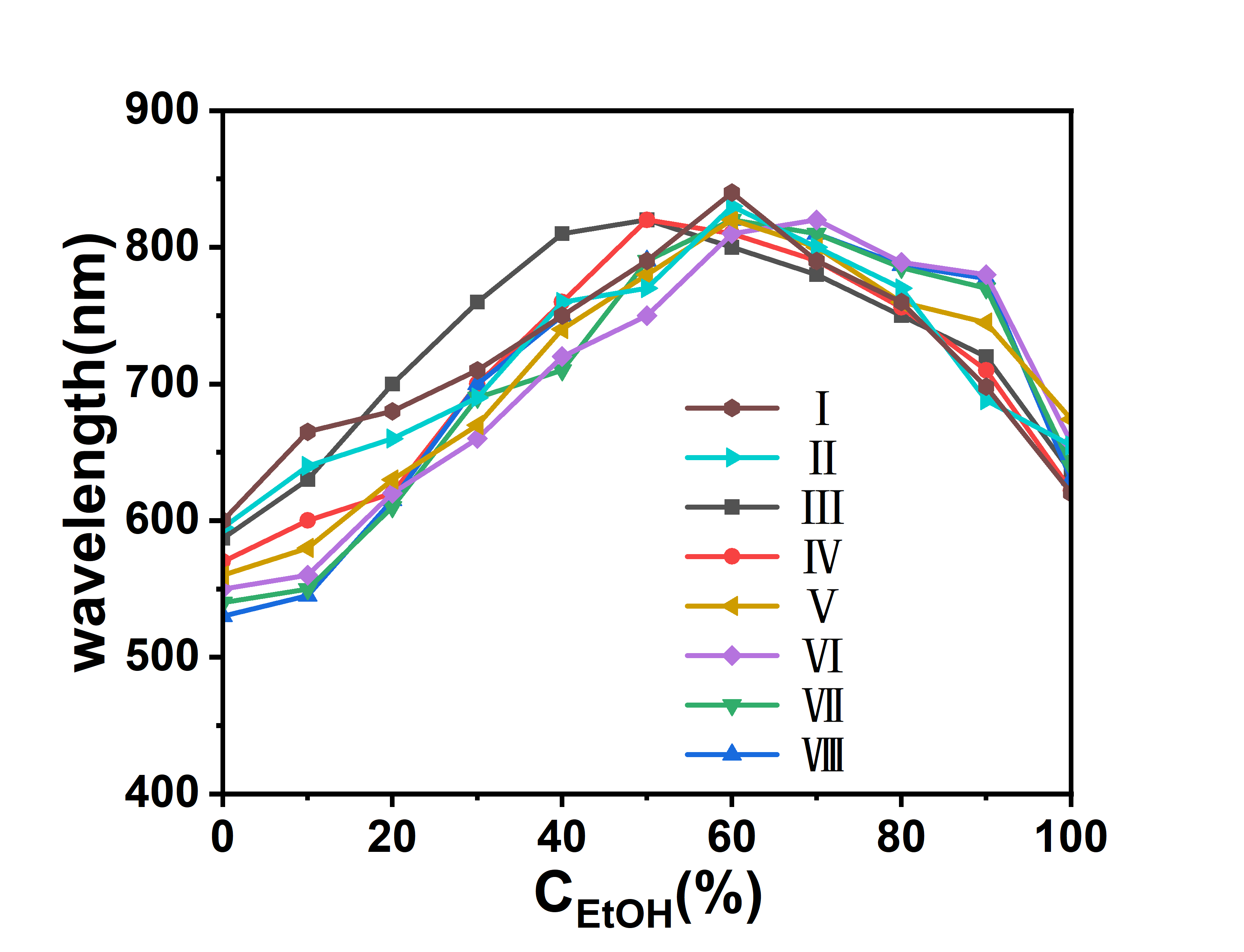


**Fig. S20** The maximum reflection wavelength of the ethanol concentration sensing array PAMBP - ethanol concentration variation curve

This work prepared a PAMBP film with a chrysanthemum pattern by irradiating it with ultraviolet light. The ultraviolet exposure time for each petal was different, as shown in Figure S7. The chrysanthemum patterned PAMBP was used to conduct response tests on alcohol-water solvents of different concentrations, and the reflection spectra of the eight petals were respectively tested, as shown in Figure S8. It was found that the structural color reflection wavelengths of the eight petals first increased and then decreased. Responding by using PAMBP structured color films with such regional differences can increase the accuracy and fault tolerance of sensing.


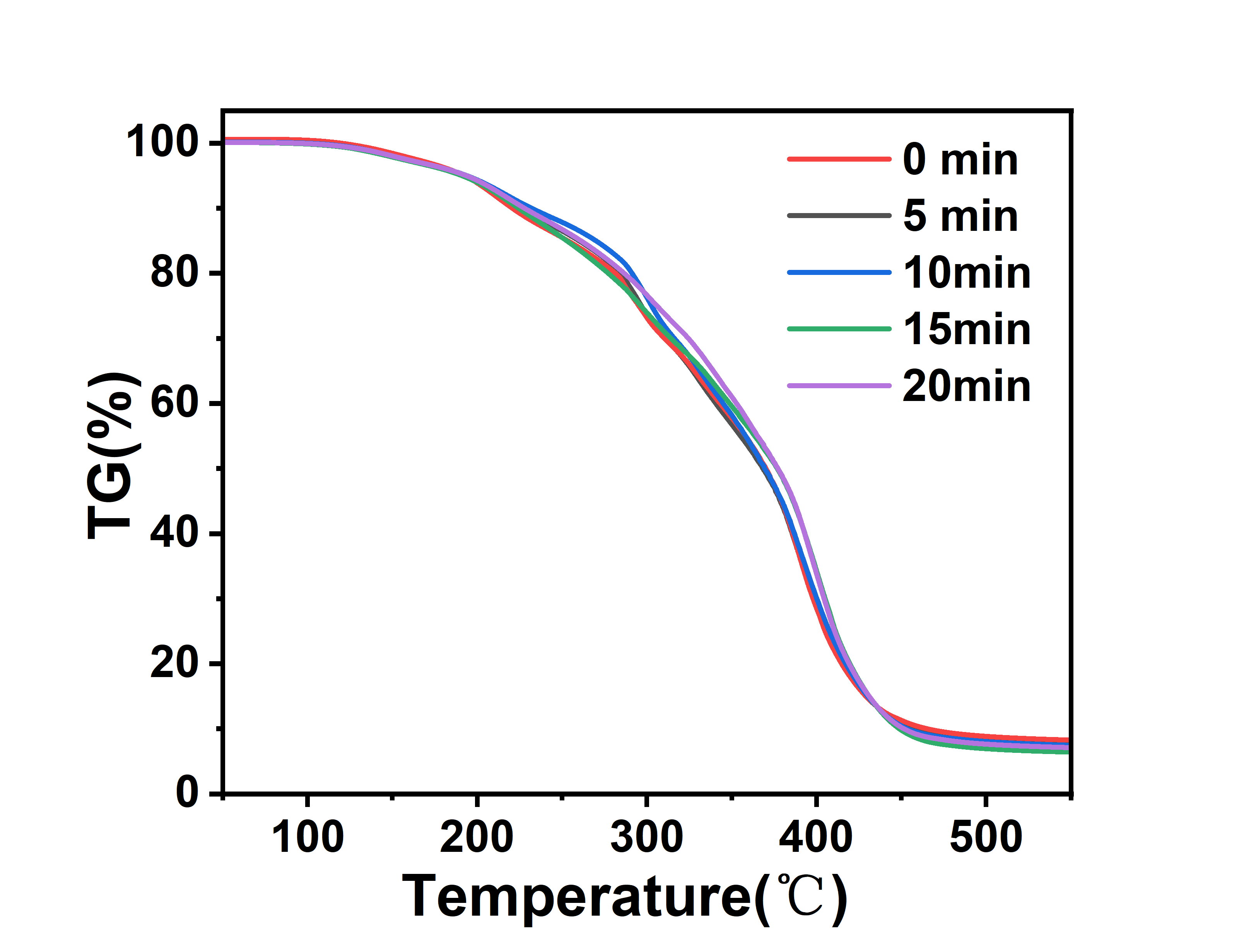

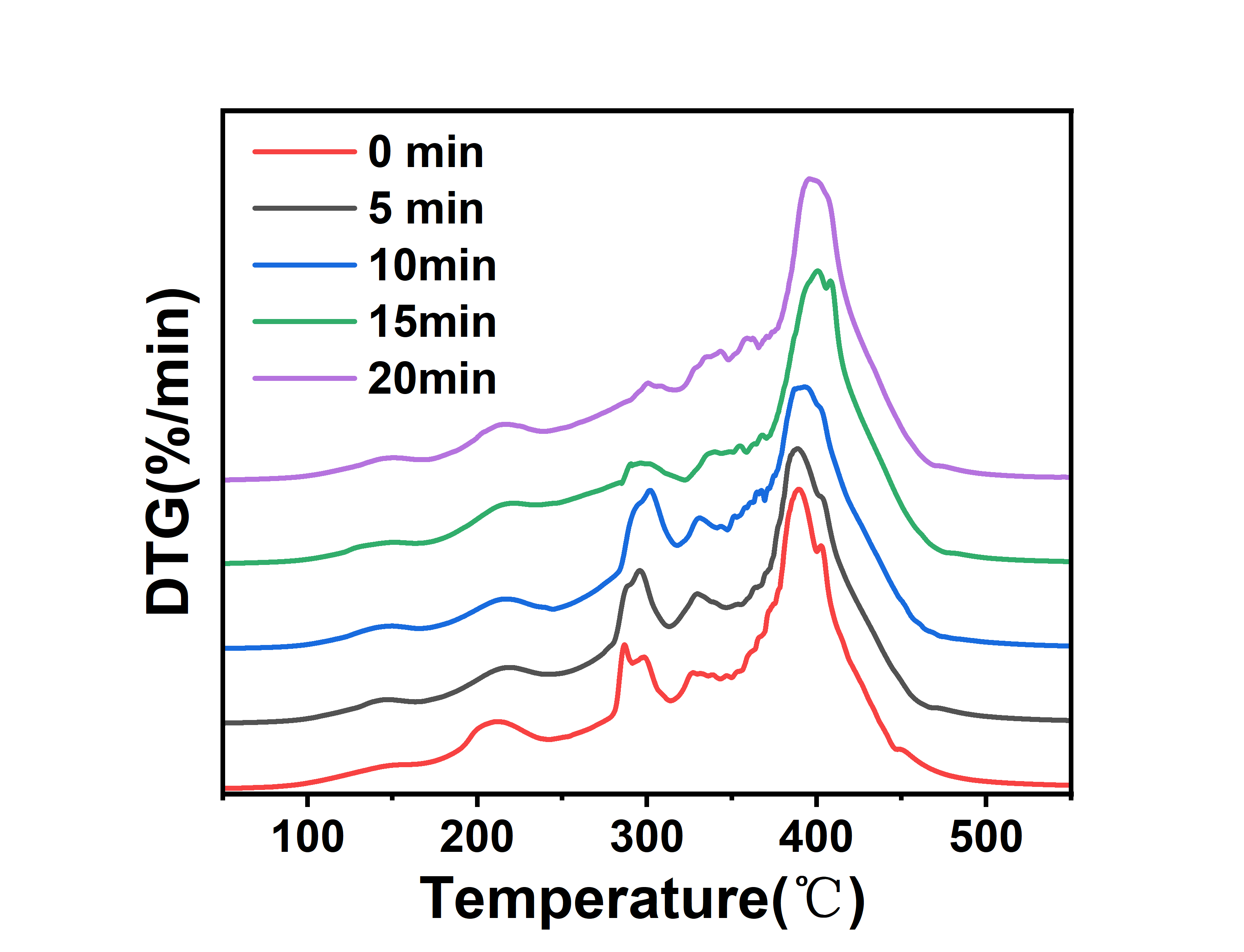


**Fig. S21** TG and DTG curves of PAMBP under ultraviolet light irradiation for different times


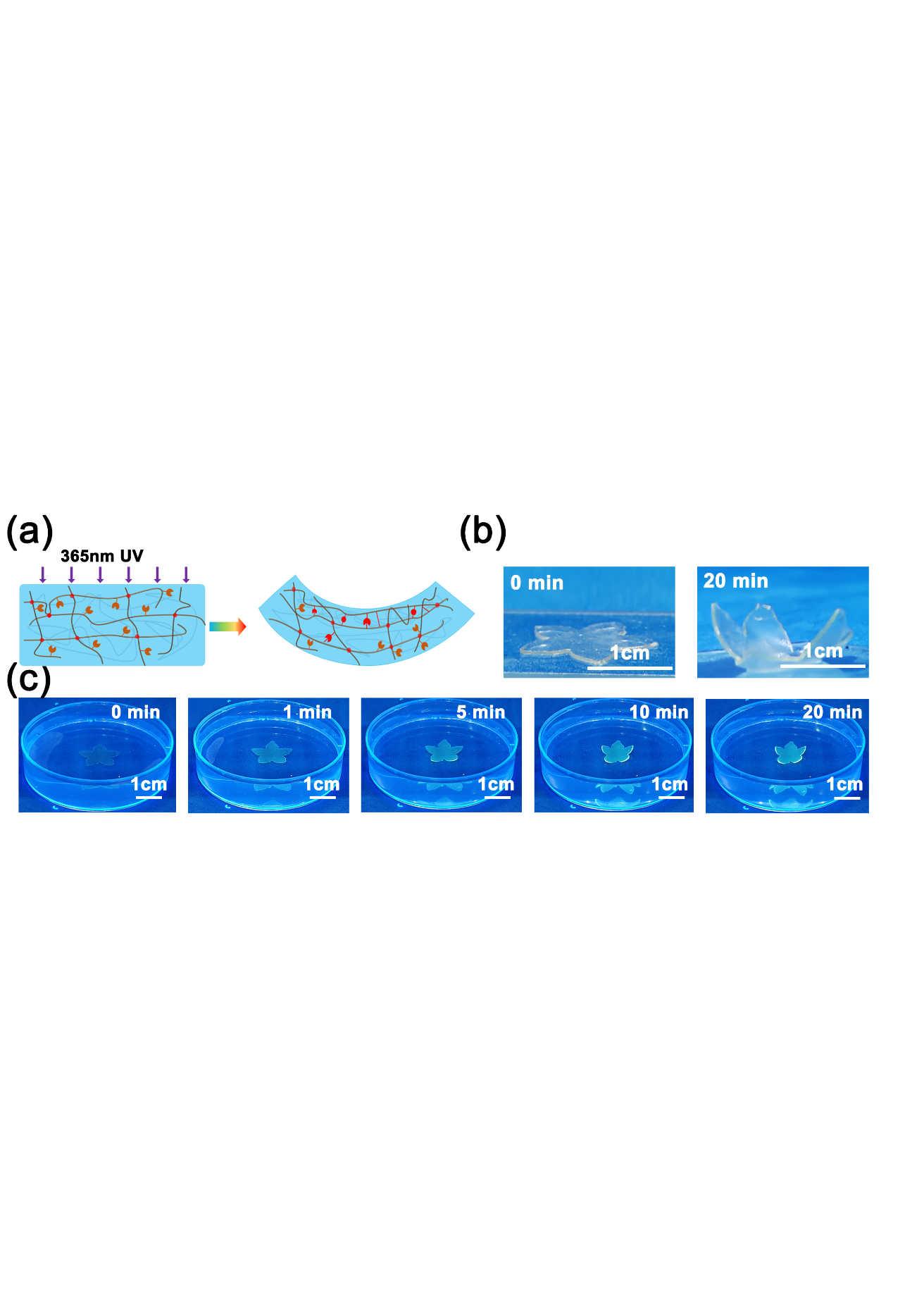


**Fig. S22** **a** Schematic diagram of the single-sided photo-curing process of PAMBP. **b**, **c** Optical photos of the closing process of light-cured flower-shaped PAMBP films


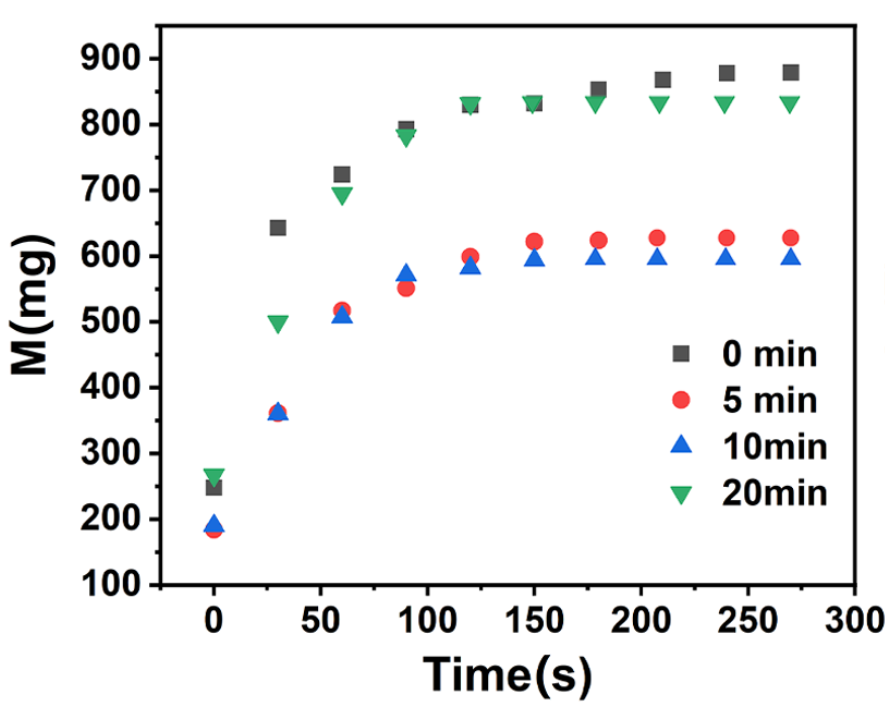


**Fig. S23** PAMBP swelling mass-time scatter plot under different-times’ UV light exposure


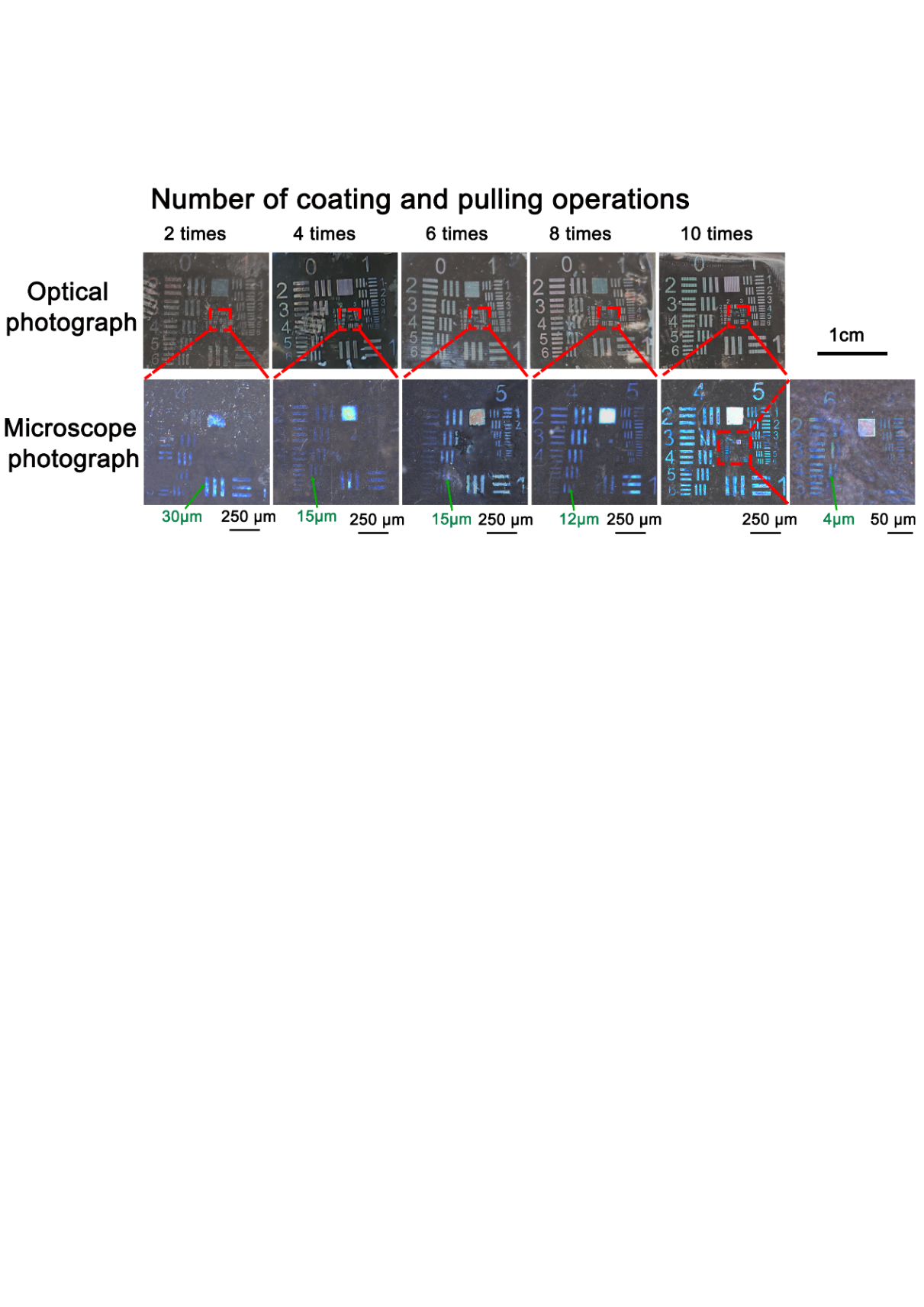


**Fig. S24** Optical and microscopic images of PAMBP (templates with different numbers of coating and pulling processes) prepared by patterning with mask


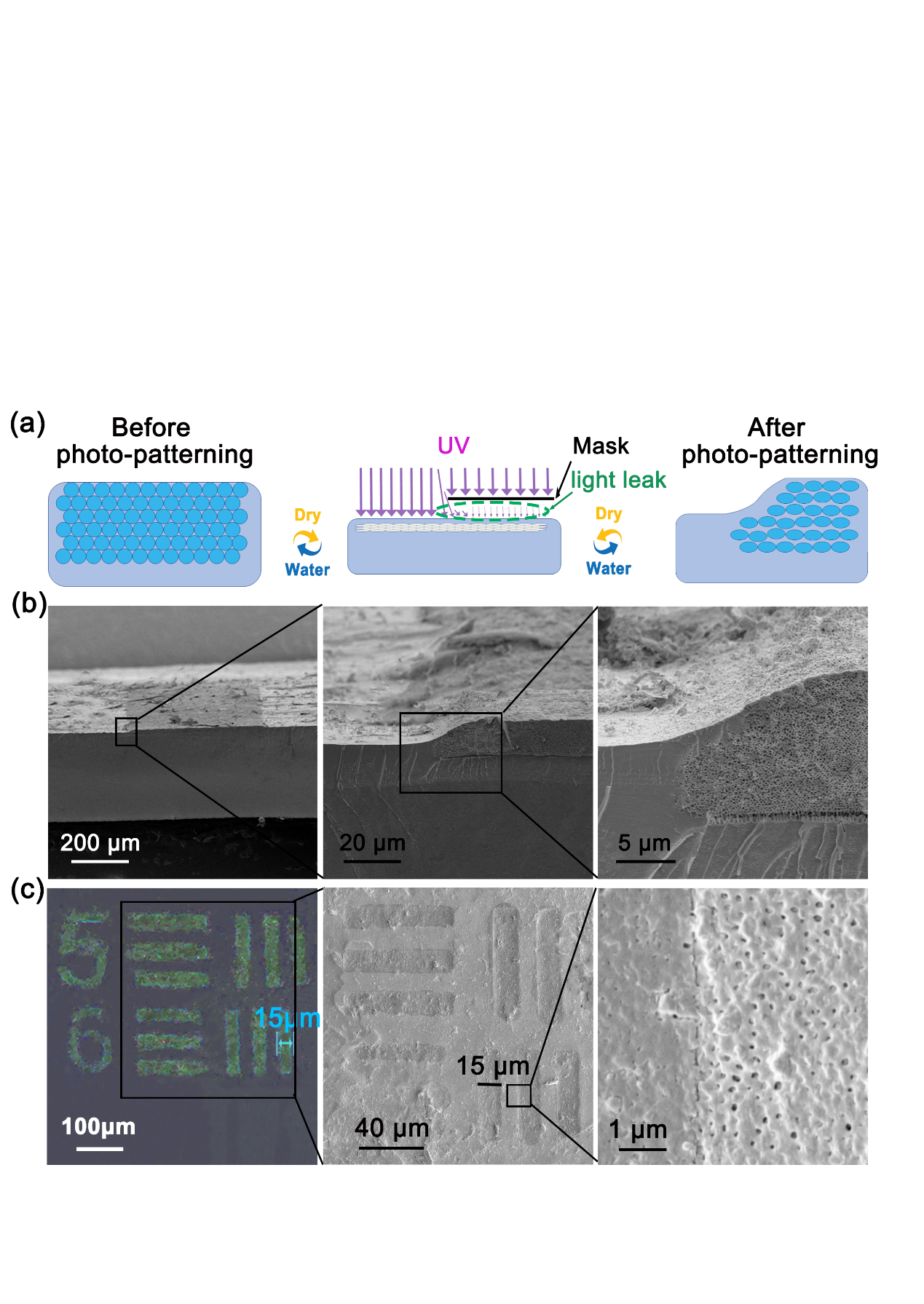


**Fig. S25** a Schematic diagram of PAMBP photo-patterning. b SEM image of the cross-section of PAMBP after photo-patterning. c Microscopic and SEM images of the surface of PAMBP after photo-patterning


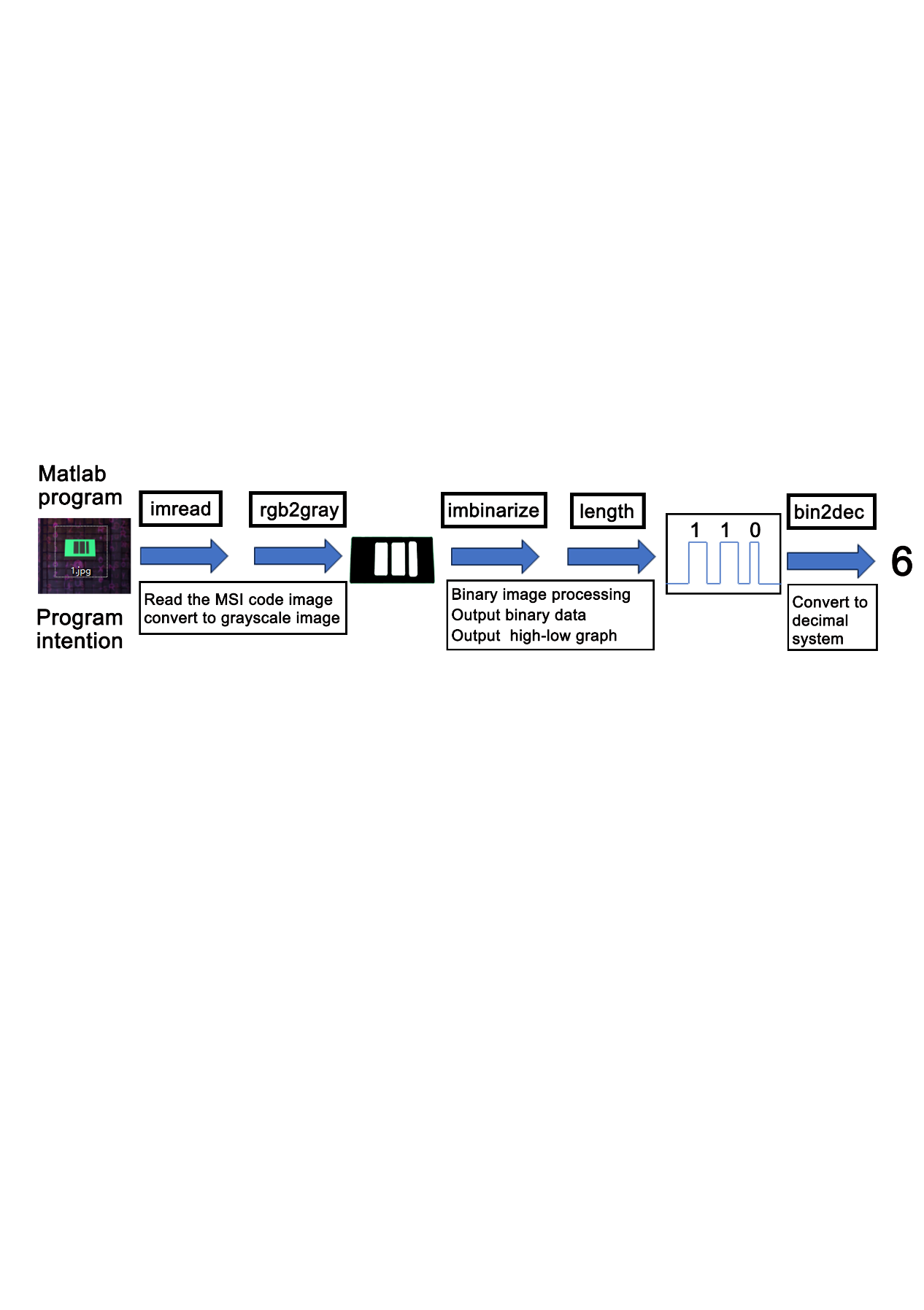


**Fig. S26** The core algorithm and flowchart of the MATLAB program script

**Supplementary References**

1. G. H. Lee, T. M. Choi, B. Kim, S. H. Han, J. M. Lee et al. Chameleon-Inspired Mechanochromic Photonic Films Composed of Non-Close-Packed Colloidal Arrays. ACS Nano **11**, 11350-11357 (2017). <https://doi.org/10.1021/acsnano.7b05885>
2. P. Vukusic. Evolutionary Photonics with a Twist. Science **325**, 398-399 (2009). <https://doi.org/10.1126/science.1177729>
3. W. Fan, J. Zeng, Q. Gan, D. Ji, H. Song et al. Iridescence-controlled and flexibly tunable retroreflective structural color film for smart displays. Sci. Adv. **5**, eaaw8755 (2019). <https://doi.org/10.1126/sciadv.aaw8755>
4. Y. Qi, L. Song, C. Zhou, S. Zhang. Hydration Activates Dual-Confined Shape-Memory Effects of Cold-Reprogrammable Photonic Crystals. Adv. Mater. **35**, 2210753 (2023). <https://doi.org/10.1002/adma.202210753>
5. P. L. Ritger, N. A. Peppas. A simple equation for description of solute release II. Fickian and anomalous release from swellable devices. J. Controlled Release **5**, 37-42 (1987). <https://doi.org/10.1016/0168-3659(87)90035-6>
